# Role of Neutrophils in Homeostasis and Diseases

**DOI:** 10.1002/mco2.70390

**Published:** 2025-09-18

**Authors:** Xingyu Chang, Yulin Liu, Junjun Qiu, Keqin Hua

**Affiliations:** ^1^ Shanghai Key Lab of Reproduction and Development Shanghai Key Lab of Female Reproductive Endocrine Related Diseases Obstetrics & Gynecology Hospital of Fudan University Shanghai China; ^2^ Cancer Center Union Hospital Tongji Medical College Huazhong University of Science and Technology Wuhan China; ^3^ Institute of Radiation Oncology Union Hospital Tongji Medical College Huazhong University of Science and Technology Wuhan China

**Keywords:** cancer, homeostasis, immunotherapy, NETs, neutrophils

## Abstract

Neutrophils, constituting a predominant subset of innate immune cells in mammalian systems, play pivotal roles in pathogenic clearance and homeostatic maintenance. In the progressive development of cancer, neutrophils exert dual roles in both anticancer and procancer processes through their heterogeneity. In recent years, research into the role of neutrophils in cancer and various nontumor diseases has been continuously deepening. However, current research in this area remains incomplete. This review comprehensively summarizes the tissue homing dynamics, lifespan regulation, and physiological functions of neutrophils, starting from their development and heterogeneity. Furthermore, we delineate the dual regulatory functions of neutrophils in carcinogenesis, encompassing both tumor‐suppressive mechanisms and protumor mechanisms. This section further synthesizes recent advancements in neutrophil‐targeted therapeutic platforms and biomimetic delivery systems, while critically evaluating persistent methodological and translational challenges in clinical applications. In addition, we systematically analyze the role of neutrophils in non‐neoplastic diseases and list several typical diseases, including infectious diseases. Finally, we also discuss current controversies and research perspectives on neutrophils. It is hoped that this review will deepen insights into the role of neutrophils in homeostasis and disease, while exploring their potential in disease treatment.

## Graphical abstract

Neutrophils are involved in the progression of various diseases through multiple mechanisms, including inflammatory responses, endothelial damage, and NETs release. (A) Cancer development and progression; (B) sepsis; (C) COVID‐19; (D) autoimmune diseases; (E) neurodegenerative diseases; (F) T2DM (e.g., diabetic foot ulcers (DFUs)); (G) nonalcoholic fatty liver disease (NAFLD).

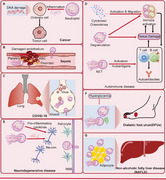

## 1 Introduction

Neutrophils originate from hepatic stellate cells (HSCs) in the bone marrow (BM), are the main component of polymorphonuclear leukocytes (PMNs), and the most abundant type of leukocyte within the human bloodstream, and are considered to be an important participatory player in the body's innate immunity [1]. Stimulated by cytokines, neutrophils are released from the BM, enter the circulatory system, and are constantly replenished and renewed. As inflammatory immune cells, neutrophils execute pathogen eradication via three distinct antimicrobial modalities: (1) phagocytic clearance, (2) granular enzyme release, and (3) neutrophil extracellular trap (NET) formation, play an important role in the body's physiological immune surveillance, immune regulation, and homeostasis [2]. Neutrophils also promote a variety of physiological functions in the body, including angiogenesis, tissue repair and body metabolism [3]. By regulating various physiological mechanisms in the body, neutrophils play an important role in maintaining homeostasis.

When homeostasis is disrupted, neutrophils also participate in disease progression. In recent years, the role of neutrophils in cancer has been further elucidated [4]. Neutrophils exhibit heterogeneity and can exert a dual effect of promoting cancer in the early stages and inhibiting cancer in the later stages through phenotypic differentiation at different stages of tumor development [5].

And neutrophils are playing an increasingly important role in other disease areas, like infectious diseases, autoimmune diseases, neurodegenerative diseases, and metabolic diseases [6, 7]. Based on the physiological functions of neutrophils themselves and their dual mechanisms (protective defense and pathological damage) in the progression of diseases, neutrophils can regulate the progression of these diseases through mechanisms such as mediating inflammatory responses, immunomodulation and regulating metabolism.

To better understand the role of neutrophils in homeostasis and disease, we provide an overview of the developmental trajectory, functional evolution, and role of neutrophils in disease. The focus is on the “variable morphology” of neutrophils during tumorigenesis and development, as well as on breakthroughs in neutrophil‐based cancer therapeutic strategies and drug delivery technologies, and the current challenges associated with clinical trials. In addition, we discuss the role of neutrophils in nontumorigenic diseases and list the corresponding typical diseases. As an important immune cell, neutrophils play an important role in body homeostasis and disease development. Therefore, research of neutrophils is necessary. This review deepens our understanding of the role of neutrophils in disease progression and provides systematic insights for the development of neutrophil‐related therapies.

## 2 Fundamental Biology of Neutrophils

Neutrophils are believed to originate in the BM, gradually acquiring phenotypic differences during development, and ultimately exhibiting functional heterogeneity under both steady‐state and pathological conditions. Most neutrophils enter the circulatory system from the BM, perform a series of physiological functions, and ultimately return to the BM for clearance. Recent studies indicate that although the lifespan of neutrophils is brief, it is regulated by multiple factors and can vary under different conditions to adapt to the body's needs.

### 2.1 Development and Heterogeneity

#### 2.1.1 Development of Neutrophils

**FIGURE 1:**
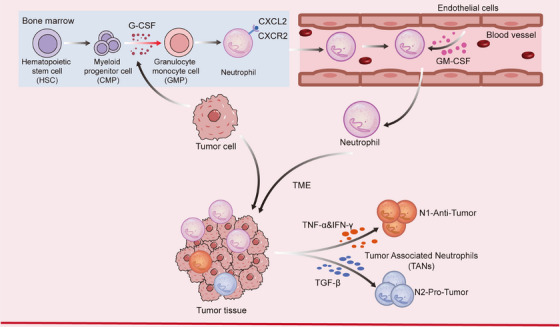
The origin, development, and differentiation of neutrophils, as well as their heterogeneity in cancer. Neutrophils develop from hematopoietic stem cells and gradually mature, eventually being released from the bone marrow into the blood. In a cancerous environment, neutrophils differentiate into two phenotypes: antitumor (N1) and protumor (N2), indicating that neutrophils exhibit heterogeneity.

Neutrophils develop from HSCs in the BM and are an important participant in the body's innate immunity. First, pluripotent granulocyte–monocyte progenitors (GMPs), which develop from myeloid pluripotent cells, give rise to neutrophil and monocyte precursors, which subsequently develop into promyelocytes and express the neutrophil lineage marker CD66b [1]. Neutrophils begin to develop from this point on, and after upregulation of CD11b and CD16 expression, promyelocytes differentiate into immature neutrophils and mature neutrophils, all of these processes occurring in the BM [1]. Mature neutrophils are continuously released from the BM into the bloodstream under the stimulation of certain specific growth factors, in particular cell colony‐stimulating factor (G‐CSF) and granulocyte macrophage colony‐stimulating factor (GM‐CSF), accounting for 50–70% of the circulating system leukocytes, and are continuously replenished and renewed [8]. In addition, the process of neutrophil mobilization in the BM is highly dependent on the regulation of the expression of genes encoding CXCR4 and CXCR2. Under homeostatic conditions, osteoblasts and other blood stromal cells express CXCL12, which signals the retention of CXCR4^+^ neutrophils within the blood stroma. During neutrophil maturation, BM and other blood stromal cells will downregulate the expression of CXCR4, which triggers the release of neutrophils into the bloodstream, while the expression of CXCR2 is upregulated on mature neutrophils, which promotes the entry of neutrophils into the bloodstream by interacting with CXCL1 and CXCL2 produced by endothelial cells (ECs) and megakaryocyte [5, 9] (Figure 1).

#### 2.1.2 Temporal Heterogeneity of Neutrophil Evolution

It has been found that neutrophils tend to follow a developmental continuum called “neutrotime” [10]. This spectrum extends from immature preneutrophils (mainly in the BM) to mature neutrophils (mainly in the blood and spleen) [11]. As neutrophils develop along this spectrum, they gradually acquire a phenotype. Neutrophils in the blood and organs show phenotypic and functional heterogeneity due to factors such as physiological state (e.g., maturation, senescence, activation), circadian rhythms, and tissue signals [12]. Using transcriptomic data, Xie et al. [13] identified up to eight neutrophil populations in the human body: G0, G1, G2, G3, G4, G5a, G5b, and G5c. These subpopulations possess different maturation states and are found mainly in the BM, spleen, and peripheral blood. They exist normally in homeostasis and are disturbed in pathological conditions such as inflammation. In contrast, there are three major neutrophil subpopulations in the peripheral blood: G5a, G5b, and G5c, which have a typical mature neutrophil nuclear morphology and are considered to be mature neutrophils with potent chemotactic, phagocytic, and bactericidal capacities [13]. During acute infectious or inflammatory responses, neutrophils undergo rapid phenotypic transformation. These cells exhibit diminished migratory capacity, reduced antimicrobial effector functions, and developmental immaturity [14, 15]. If the inflammatory stimulus persists, acute injury will lead to matrix remodeling and myelopoietic activation, inducing the production of myelopoietic factors (such as G‐CSF) and consequently granulopoiesis [15].

Under homeostatic conditions, neutrophils are released from the BM, recirculate in the peripheral blood to reinfiltrate tissues and are removed from the bloodstream. Both these processes occur at a circadian rhythmic frequency. Although neutrophils are in the bloodstream for a relatively short period of time, their phenotype and function do undergo significant diurnal changes, a phenomenon known as neutrophil aging [16]. That means that neutrophils adjust their functions to the changing needs of the day. For example, it was found that neutrophils during an animal's active period prevented microbial invasion, while performing repair functions when the animal was at rest [17]. While neutrophils undergo immune senescence throughout their physiology, senescence can be mediated through the secretion of a range of cytokines, chemokines, growth factors, and proteases that can cause cells to possess a senescence‐associated secretory phenotype (SASP) [18, 19]. Therefore, there is also a specialized type of neutrophil that expresses SASP. SASP consists of approximately 75 secreted factors, including GM‐CSF, IL‐6, IL‐8, and IL‐10, growth factors (including HGF and IGFBP), and exfoliative cell survival factors (including ICAMs and UPARs) [20, 21, 22, 23]. Zhang et al. [24] showed that the senescent phenotype of neutrophils is triggered by the microbiota through signaling pathways mediated by Toll‐like receptor (TLR) and myeloid differentiation factor 88. In contrast, microbiota‐mediated neutrophil aging is associated with a proinflammatory phenotype. The enhanced activation of αMβ2 integrins and formation of NETs under inflammatory conditions suggests that the microbiota plays a facilitating role in the formation of proinflammatory neutrophil subpopulations.

#### 2.1.3 Spatial Heterogeneity of Neutrophil Evolution

When neutrophils encounter environmental changes during development, their development is biased by site, stimulus, and time, such as tissue‐specific transcriptional profiles and enhanced inflammatory capacity, and ultimately result in different phenotypes, which are also relevant to the microenvironment in which the cells live [11, 25]. Traditionally, it has been assumed that neutrophils are absent from healthy tissues in the absence of inflammatory injury. However, under physiological conditions, neutrophils are found in the lungs, liver, and spleen, suggesting that different subpopulations of neutrophils exist in these tissues [6, 26]. The acquisition of tissue‐specific phenotypes suggests that neutrophils may be activated differently depending on tissue‐derived signals. In the lung, large numbers of neutrophils are marginalized in the pulmonary microcirculation through CXCR4‐mediated rapid responses to foreign microorganisms [27]. In addition, neutrophils in the marginal zone of the human spleen have been identified as B‐cell helper neutrophils (NBH cells), which can promote B‐cell proliferation and antibody production through the secretion of cytokines, chemokines [28].

The spatial heterogeneity of neutrophil evolution can also be characterized on the basis of sedimentation properties. It has been found that circulating neutrophils can be categorized into high‐density neutrophils (HDNs) and low‐density neutrophils (LDNs) as revealed by density gradient centrifugation [29]. HDNs are considered regular mature neutrophils and predominate in healthy individuals [30, 31]. After stimulation, HDNs undergo degranulation accompanied by an increase in cell size and eventually transform spontaneously into LDNs in a TGF‐β‐dependent manner [32]. This transformation may be mediated by cytokines or chemokines in advanced disease, suggesting neutrophil plasticity. In contrast to HDNs, LDNs have reduced phagocytic activity, reduced migratory capacity, loss of antitumor properties, and enhanced immunosuppressive function, with characteristics opposite to those of mature HDNs [33]. LDNs can be categorized as mature or immature based on the morphology of their nuclei. Immature LDNs are less immunogenic than mature neutrophils from healthy organisms, but they can still exert chemotaxis, phagocytosis, sterilization, and reactive oxygen species (ROS) production [34, 35, 36]. It has been shown that inflammatory signals (e.g., G‐CSF, IL‐6) promote BM to release immature neutrophils prematurely [29]. Depending on their function, LDNs can be categorized into (i) immunosuppressive LDNs, also known as granulocyte myeloid‐derived suppressor cells (G‐MDSCs); (ii) proinflammatory LDNs, also known as “low‐density granulocytes” (LDGs); and (iii) immature neutrophils [37]. Among them, immunosuppressive LDNs/G‐MDSCs exerted their immunosuppressive effects mainly through the overproduction of arginase 1 (ARG1) and ROS, the PD‐L1/CD274‐dependent interaction, and the induction of CD4^+^FoxP3^+^ T regulatory (Treg) cells [38, 39, 40, 41]. In addition, in recent years, LDNs in autoimmune diseases such as systemic lupus erythematosus (SLE) have been found to have proinflammatory effects [42]. LDGs act by releasing proinflammatory factors such as tumor necrosis factor‐α (TNF‐α) and NETs [43, 44, 45, 46].

In conclusion, neutrophils differentiate into different subpopulations under the influence of the organism's microenvironment and various cellular mediators during development. Due to their heterogeneity, these subpopulations embody a variety of phenotypes and functions, and play important roles in maintaining body homeostasis and regulating disease progression. Exploring the heterogeneity of neutrophils is an important part of neutrophil research, which helps us to further understand the process of dynamic changes in body health and disease. And the disease treatment strategies carried out based on neutrophil subpopulations also provide new concepts for disease treatment. In addition, as an important manifestation of neutrophil heterogeneity in pathological states, the immature phenotype and strong immunosuppressive function of LDNs may make them key mediators hindering disease treatment. Meanwhile, in view of the proinflammatory effects of LDGs in autoimmune diseases, it may be possible to consider LDGs as a starting point for interfering with the disease process of autoimmune diseases.

### 2.2 Tissue Homing Dynamics

Once maturation is complete in the BM, neutrophils enter the blood circulation. Neutrophils have a preformed set of adhesion and chemotactic receptors and effector proteins in circulation to rapidly migrate and respond to a variety of microbial and sterile challenges. In response to inflammatory and chemotactic factors, neutrophils are progressively prepared to enter specific tissues. The process of neutrophil extravasation across the vessel wall is divided into five steps, including tethering, rolling, adherence, crawling, and migration [47]. This process is closely related to adhesion molecules such as P‐selectin and E‐selectin expressed on vascular ECs, as well as PSGL1 and l‐selectin expressed on neutrophils. In addition, molecules such as ICAM1 are also involved [5, 47, 48]. Once neutrophils are tightly bound to vascular ECs, they are transferred from the circulation to inflamed tissues and function depending on the inflammatory environment or tissue. Inflammatory tissues release chemokines that induce neutrophil aggregation. In the tumor microenvironment (TME), on the other hand, neutrophil recruitment can promote an increase in a variety of cytokines and chemokines, further altering the TME and promoting tumor development. For example, tumor‐derived G‐CSF can promote homing of neutrophils to nonadjacent tissues and promote tumor migration [49]. In addition, it has been shown in mice that platelets can induce neutrophils to move toward the site of exocytosis [50], demonstrating that platelets act as a mediator between neutrophils and activated ECs and are involved in the inflammatory response, a mechanism of neutrophil recruitment specific to mammals [51]. Also, it has been found that platelets can participate in neutrophil recruitment by regulating the expression of ICAM‐1 in ECs [52]. In addition to the BM, neutrophils are also present in organs that are in homeostasis, such as the spleen and liver in mice [53], which are sites of extramedullary hematopoiesis, but studies on extramedullary granulopoiesis are still unclear.

### 2.3 Functional Toolbox

As inflammatory immune cells, neutrophils tend to destroy pathogens through phagocytosis, degranulation, and formation of NETs. In inflammatory conditions, neutrophils undergo rapid changes in various phenotypes and functions. For example, neutrophils take different forms to migrate across the vessel wall, express a range of pattern recognition receptors, and secrete different types of cytokines involved in the activation of T‐cell capacity during infection [15]. In the early stages of inflammation, neutrophils play a dominant role. However, if the inflammatory stimulus persists and the neutrophils become depleted, the injury will lead to matrix remodeling and activation of myelopoiesis. Inflammation induces the production of myeloid hematopoietic factors such as G‐CSF and GM‐CSF, which triggers an “emergency granulopoiesis (EG)” program that promotes rapid maturation of neutrophils. This behavior results in the release of large numbers of immature neutrophils into the bloodstream in addition to mature neutrophils [54].

In tumors, high neutrophil expression of CXCR1/CXCR2 and certain inflammatory mediators such as TNF‐α together promote their recruitment into the TME. In turn, cancer cells are also involved in the phenotypic shift of neutrophils within the TME to cancer‐associated neutrophils (tumor‐associated neutrophils [TANs]) [55, 56, 57, 58]. TANs can directly promote the development of tumors by inducing tissue damage and gene mutations, or indirectly drive malignant transformation and promote tumor growth and metastasis through the production of ROS, tumor‐promoting miRNAs and other substances [9, 59]. It is worth noting that the effect of ROS depends on concentration and microenvironment. Therefore, its effects are not static. For example, cancer‐inhibiting N1‐type TANs can trigger apoptosis through the production of ROS and NO, which in turn kills cancer cells directly [60, 61]. In contrast, cancer‐promoting N2 type TANs will indirectly promote tumor development by inducing angiogenesis, promoting immunosuppression and extracellular matrix (ECM) remodeling [9].

In summary, the functional toolbox of neutrophils is a multilayered immune defense system. Through the multiple functions of phagocytosis and sterilization, granule release, NETs trapping, inflammation regulation, and metabolic burst (ROS generation) that work in concert in space and time, neutrophils can rapidly clear pathogens and regulate the immune response, constituting a fast‐responding innate immune defense. During disease progression, neutrophils also adjust their functional status according to the state of the microenvironment and participate in the progression of the body's disease process.

### 2.4 Lifespan Regulation

The lifespan of neutrophils is short and highly dynamic and is regulated by a combination of microenvironmental signals, cell‐intrinsic programs, and pathological states, with survival times ranging from a few hours in homeostasis to a few days in inflammatory states. Under physiological conditions, the transfer of newly formed neutrophils from the BM to the circulation lasts approximately 2.6 days in mice and 6 days in humans [62]. Once released into the bloodstream, the half‐life of circulating mature neutrophils is less than 1 day [63]. Traditionally, neutrophils in homeostasis are thought to have a short half‐life of 6–18 h, while the average lifespan of a neutrophil is 24 h [62]. After full effect, neutrophils are eliminated by the body's clearance mechanisms to protect the host from other than inflammatory damage.

Normally, neutrophils enter the blood circulation according to a circadian rhythm. Neutrophils in the circulation act as antimicrobial cells to help the body remove pathogens and maintain body homeostasis. Neutrophil‐mediated inflammatory response ends with apoptosis at the site of inflammation. However, neutrophils have a short lifespan. After full effect, neutrophils in the circulation gradually apoptosis and migrate to the lungs, liver, spleen, and BM, where they are cleared by a variety of routes. On the one hand, macrophages residing in various tissues and organs can directly phagocytose apoptotic neutrophils in peripheral tissues, thus preventing the loss of neutrophil contents and the resulting tissue damage. On the other hand, CXCL12, a ligand for CXCR4, also plays an important role in mediating neutrophil clearance, and circulating neutrophils are recruited back to the BM for clearance via the CXCR4/CXCL12 axis [64]. In addition, it has been found that neutrophils can also die through autophagy, necrosis and phagocytosis. And NETs themselves are a result of neutrophil lysis and death, known as NETosis [65]. Depending on the surrounding environment, neutrophils undergo different types of cell death. Because neutrophils have a short lifespan and are unable to proliferate, they need to rely on this constantly renewed clearance mode of operation, and normally, when the internal environment is stable, circulating neutrophils are in a state of balanced release and clearance.

A number of factors are involved in regulating the lifespan of neutrophils as they function. Numerous studies have shown that the absence of antiapoptotic proteins is a major mediator of spontaneous neutrophil apoptosis. The antiapoptotic proteins Mcl‐1 and A1 belong to the antiapoptotic Bcl‐2 family and regulate neutrophil survival and homeostasis through the PI3K/Akt and JAK/STAT pathways, Overexpression of Mcl‐1 and A1 inhibits neutrophil apoptosis. Under homeostasis, the synthesis of Mcl‐1 and A1 is reduced and rapidly degraded by the proteasome, accelerating neutrophil apoptosis [66]. Activation of the proapoptotic protein Bax/Bak leads to disruption of mitochondrial membrane integrity and release of cytochrome *C*, which promotes intrinsic activation of caspase‐9, leading to apoptosis. Caspase‐3 is activated in the spontaneous apoptotic pathway of neutrophils, and inhibiting either caspase‐3 or caspase‐8 significantly delays neutrophil apoptosis [67]. In addition, metabolism also limits neutrophil survival. The metabolism of neutrophils is dominated by glycolysis, and the accumulation of mitochondrial ROS promotes DNA damage and apoptosis.

Neutrophils circulate in the bloodstream for 1–2 days before they die by apoptosis and are eliminated. However, if neutrophils are recruited to the site of infection, the lifespan of neutrophils is greatly extended in the presence of proinflammatory factors. The proinflammatory factors G‐CSF, TNF‐α, IL‐8, and so on and stimulants such as LPS in the inflammatory environment can prolong the lifespan of neutrophils through various pathways such as JAK/STAT3, NF‐κB, and PI3K/Akt, leading to delayed apoptosis of neutrophils [68, 69, 70, 71, 72]. As a homeostatic mechanism, as inflammation subsides, senescent neutrophils undergo passive apoptosis due to the scarcity of proinflammatory factors. This process involves Fas/CD95/Apole signaling, while spontaneous apoptosis of neutrophils does not require signaling through Fas. Therefore, the production of proinflammatory factors at the site of inflammation delays neutrophil apoptosis, which facilitates the elimination of pathogens. But in the absence of infecting pathogens, this would result in the persistence of neutrophils at the site of inflammation and nonspecific tissue damage [73]. Autophagy is an intracellular degradation mechanism designed to maintain homeostasis in the intracellular environment. In response to autophagy, damaged organelles are transported to lysosomes for degradation [74]. In the early stages of infection, autophagy prolongs the lifespan of neutrophils by removing damaged mitochondria, reducing the accumulation of ROS, and protecting neutrophils from cytokine‐induced stress such as G‐CSF. However, when cells are under stress, excessive autophagy leads to autophagic cell death, which promotes the formation of NETs and thus induces chronic inflammation [73]. Neutrophils may undergo reverse migration under the regulation of the tissue microenvironment. Hughes et al. [75] found that in a mouse model of glomerular capillary injury, the majority of neutrophils entering the inflamed glomerular capillaries did not undergo apoptosis and were able to re‐enter the circulatory system. This process by which neutrophils at the site of injury escape apoptosis and migrate backward into the vascular system has been termed retrograde trans endothelial migration of neutrophils [76], This process may be associated with activation of hypoxia‐inducible factor 1‐α (HIF‐1α), reduction of CXCR1, and chemotaxis of certain lipid mediators (e.g., LTB4). During this process, neutrophils have an extended lifespan, relocalize, and may re‐engage in organismal immunosurveillance [77].

The lifespan of neutrophils is a plastic and dynamic process, regulated by a precise balance of microenvironmental signals and cell‐intrinsic networks. Targeting lifespan regulatory pathways (e.g., Mcl‐1 degradation, PI3K/Akt pathway inhibition, and modulation of cytokines such as HIF‐1α and GM‐CSF) is expected to provide novel therapeutic strategies for tissue infections, cancers, and autoimmune disorders by regulating the survival time of neutrophils and promoting further neutrophil function.

## 3 Neutrophils in Homeostasis

The functions of neutrophils under steady‐state conditions can be divided into three main parts: physiological immune surveillance, tissue repair, and metabolic regulation. Under homeostasis, neutrophils continuously perform immune surveillance functions. When pathogens are identified, neutrophils defend the body through three classic effector functions: phagocytosis, degranulation, and release of NETs. At the same time, neutrophils also participate in the repair of damaged tissues and the regulation of metabolic energy to maintain or restore homeostasis.

### 3.1 Physiological Immune Surveillance

Neutrophils, as important immune cells of the organism, bear a considerable immune function. Under homeostasis, neutrophils are not quiescent, but actively perform immunosurveillance functions. They maintain tissue homeostasis by dynamically monitoring the tissue microenvironment and removing potential threats. Neutrophils are the first line of defense in the immune response. Under chemotaxis, neutrophils continuously patrol the circulation and various tissues, recognizing pathogens through surface receptors [7]. The three classical effector functions of neutrophils include phagocytosis, degranulation, and release of NETs.

When neutrophils recognize trace pathogens or damage signals, neutrophils are activated and initiate a molecular cascade that recruits additional immune cells to the infected area as well, joining the defense team. Neutrophils can induce phagocytosis by recognizing protein signals wrapped around pathogens via cell membrane receptors and forming phagosomes [78]. On the mucosal surfaces of the intestinal and respiratory tracts, neutrophils are involved in the formation of cellular defense barriers, and through phagocytosis, they eliminate invading or colonizing potential microbial pathogens and prevent them from proliferating excessively or breaking through the mucosal barriers, which can damage the organism [79, 80].

If phagocytosis alone fails to contain the infection, neutrophils activate multiple pathways through phagocytosis, in which phagosomes produce ROS via NADPH oxidase (NOX) and degranulate to release bactericidal proteins that in turn kill the pathogen [81]. Mature neutrophils possess three types of granules, each releasing a different protein in sequence [47, 82]. The first to be released are tertiary granules such as ARG1 and matrix metalloproteinase 9 (MMP9). This is followed by secondary granules that release proteins, including lactoferrin, MMP8, β2‐microglobulin, and hemoglobin. Finally, there are primary granules (also called nitrogen‐loving granules), which release a variety of proinflammatory and antimicrobial proteins, including myeloperoxidase (MPO), neutrophil elastase (NE), defensins, serine proteases, proteinase 3, thrombin G (CG) and C, and bactericidal permeability‐increasing protein [83]. NOX‐generated superoxide also activates MPO, which is released from nitrogen‐loving granules while promoting the release of NE, and MPO and NE can act cooperatively and cleave cells [84, 85]. NE can degrade damage‐associated molecular patterns (DAMPs) such as proinflammatory factors, alarmins, HMGB1, and heat shock protein 90 to promote inflammation, thereby interfering with the recruitment of other inflammatory cells [86, 87, 88].

Although phagocytosis and degranulation are the primary defense mechanisms of neutrophils, when pathogens are too large to be phagocytosed, neutrophils are activated and release NETs to physically capture and kill the pathogen [83]. In 2004, Brinkmann et al. discovered that neutrophils undergo a unique type of caspase‐independent cell death called NETosis, which releases a meshwork decorated with depolymerized chromatin DNA and antimicrobial molecules, a structure that has been named neutrophil extracellular entrapment networks (NETs) [54, 89]. Factors that stimulate neutrophils to trigger NETosis include (i) bacteria and bacterial component LPS, (ii) cytokines such as IL‐8, TNF‐α, and (iii) endogenous metabolites such as linoleic acid, hyperglycemia, and cholesterol [90]. Through in vitro induction experiments, they further demonstrated that NETs can resist virulence factors and kill bacteria. Also, they found that bacteria and fungi and so on can also stimulate the organism leading to the release of NETs. Three types of NETosis have been identified, including suicidal NETosis, viable NETosis, and mitochondrial NETosis [91, 92].

NETs not only provide a high concentration of antimicrobial substances locally, but also act as a physical barrier to impede the spread of pathogens. As a host defense mechanism, NETs can effectively protect against viral and fungal infections [93]. In addition, neutrophils can activate local immune responses by releasing inflammatory mediators such as NETs or IL‐8, which combine with other immune cells to form an early immune defense system. NETs can efficiently capture and destroy a large number of microorganisms and mark the infected area and activate adaptive immunity, which facilitates the immune response of other immune cells [94, 95]. At the same time, neutrophils produce calreticulin, which activates the alternative complement pathway and releases C5a to clear invading pathogens. Moreover, the complement pathway in turn leads to further activation of neutrophils [96].

In conclusion, neutrophils play an important role in the homeostatic balance of the organism by participating in immune surveillance and defense through three major mechanisms: phagocytosis, degranulation, and release of NETs, and further activating the body's immune response through the activation of local immune responses and cascade adaptive immunity.

### 3.2 Tissue Repair Function

In addition to their primary immune function, neutrophils are also involved in tissue repair in the body. It has been found that neutrophils are often the first to be recruited to the wound site when tissue is injured. Neutrophils can phagocytose and eliminate inflammatory debris, initially purifying the wound and laying the initial foundation for wound healing and tissue repair [97, 98]. Studies have shown that NE can induce fibroblast proliferation and myofibroblast differentiation [99]. Meanwhile, the functions of phagocytosis, ROS generation, degranulation, and NETs of neutrophils also prevent microbial growth. It has been found that when the CXCR2 gene was knocked down, neutrophils could not be recruited to the wound, thus delaying wound healing [100].

In addition, neutrophils can promote angiogenesis. The CXCR4/CXCL12 signaling axis has been shown to be associated with vascular repair under homeostasis and inflammation. MMPs also degrade the ECM and release matrix‐bound vascular endothelial growth factor (VEGF), which has a proangiogenic function. While neutrophils can secrete MMP9 and are unaffected by its inhibitors, they can also transport MMP9 to angiogenic sites [101]. At the same time, MMPs can regulate matrix–cell and cell–cell interactions by cleaving structural proteins such as collagen in the ECM, ultimately leading to loosening of cell–matrix attachment and providing a basis for cell migration [102]. Furthermore, neutrophils can transport perivisceral matrix to the site of injury, promoting tissue repair and establishing new ECM at the wounds [103]. In addition to transporting pre‐existing ECM, neutrophils can also actively generate ECM under certain specific conditions. For example, during myocardial infarction, neutrophils promote ECM deposition in the ischemic heart by producing fibronectin and fibrinogen [104].

It has been proposed that another important mechanism by which neutrophils promote tissue repair is the activation of a macrophage‐centered feedforward program that in turn promotes tissue repair. Apoptotic neutrophils phagocytosed by macrophages release interferon (IFN)‐β, TGF‐β, and IL‐10 that promote tissue repair [105, 106, 107]. In addition, it has been shown that neutrophils can replace CCR2^+^ macrophages as the main phagocytes in neuroinvasive diseases and initiate neuronal repair by removing myelin debris through phagocytosis after peripheral nerve injury [108]. It is thus clear that after the anti‐infective effect, the role of neutrophils is gradually shifted to repair, and their action is precisely regulated by the local microenvironment and cellular interactions (e.g., macrophages) for the homeostasis of the organism.

### 3.3 Metabolic Regulation Function

Compared with other immune cells, neutrophils have fewer and smaller mitochondria and are relatively less active, and the ATP produced by the TCA cycle is insufficient for energy supply [109, 110]. Therefore, neutrophils rely primarily on anaerobic glycolysis to generate ATP and produce NADPH to provide energy for processes that depend on NADPH oxidation (e.g., ROS generation and NETosis). Thus, a large part of the effect of neutrophils depends on glucose metabolism. In addition, it has been found that neutrophils also utilize amino acids and lipids to produce energy.

Under homeostatic conditions, neutrophils are preferentially supplied with energy via mitochondrial oxidative phosphorylation (OXPHOS) to maintain a low metabolic state for prolonged lifespan. Under inflammatory or infectious conditions, a large number of neutrophils are required to regulate effector functions via cellular metabolism, and thus neutrophils have elevated energy requirements and need to rely on anaerobic glycolysis for additional ATP production [111]. When the onset of inflammation, cancer and autoimmune diseases lead to the activation of large numbers of neutrophils, the metabolic demands on neutrophils to perform the processes of phagocytosis, degranulation, and the role of NETs are heightened, and neutrophils intensify their glucose uptake, with shifts in metabolic profiles including enhanced anaerobic glycolysis, mitochondrial alterations, and shifts to fatty acid and amino acid metabolism, ultimately leading to a heavy metabolic shift in neutrophils [112, 113]. These metabolic changes are regulated by key signaling pathways, including HIF‐1α, NF‐κB, and mTOR [114, 115, 116, 117]. For example, hypoxic conditions such as inflammation and cancer induce the release of HIF‐1α, which can contribute to metabolic reprogramming of neutrophils toward glycolysis and other anaerobic processes [118].

When glycolysis is inhibited, the bactericidal effect of neutrophils is significantly reduced [119]. In addition, glycolysis produces energy under both aerobic and anaerobic conditions, which is very important in inflammatory tissues [120]. Furthermore, neutrophils utilize glucose for NADPH production, ROS generation, and NETs formation for energy [121]. And this is supported by the increased tendency of neutrophils to develop NOX‐dependent NETs in diabetic patients [122, 123]. Moreover, ROS also promote neutrophil glycolysis in turn [119]. Phagocytosis and the stimulation of LPS also lead to the accumulation of lactic acid, which is a product of glycolysis, and lactic acid can act as an antimicrobial molecule to kill bacteria directly, and also increase the activity of CD8+ T cells to promote a cellular immune response [124, 125]. While the upregulation of pyruvate kinase M2 expression during glycolysis will also regulate the migration ability of neutrophils through phosphorylation and participate in the formation of NET [111]. In conclusion, glycolysis plays an important role in the metabolic regulation of neutrophils.

It has been found that under acute conditions such as inflammation, neutrophils not only generate energy through glycolysis, but also generate glucose by upregulating the utilization of nonglucose substrates such as glutamine or palmitate to provide energy for further effects [126]. Glutamine supplies energy to mitochondria via the TCA cycle, promotes the production of TNF‐α, and provides an electron transport chain substrate for ROS production [127, 128]. In inflammation, enhanced glutamine metabolism increases the bactericidal function of neutrophils. Neutrophils also inhibit T‐cell proliferation and function by depleting arginine. Neutrophils also produce NADPH via the pentose phosphate pathway, which accelerates the production of ROS in response to stimuli and is used to kill pathogens [129, 130]. A growing number of studies have pointed out that lipids also play a pivotal role in the normal development and functioning of neutrophils. Energy generated from lipid metabolism is mainly derived from mitochondrial β‐oxidation [131]. Aggregated lipid droplets in neutrophils might be related to their phagocytosis [132, 133]. In addition, neutrophils express the low‐density lipoprotein (LDL) receptor (LDLR) and internalize LDL, which is also associated with cholesterol synthesis [134].

In conclusion, neutrophils participate in physiological immune surveillance and immune regulation through the secretion of various cytokines and the production of NETs. In addition, neutrophils maintain homeostasis by regulating physiological processes such as tissue repair and metabolic regulation. When homeostasis is disrupted or imbalanced, or even when disease is induced, neutrophils are also involved in disease progression.

## 4 Neutrophils in Cancer: A Stage‐Dependent Paradox

Cancer initiation and development are not only influenced by inherited factors such as genes, but also depend largely on the external environment, that is, the TME, which consists of tumor cells, stromal cells, ECs, a variety of immune cells, and components of the ECM, and is a kind of local inflammatory microenvironment that is conducive to the development, progression, and metastasis of tumors. With the deepening of related studies in recent years, it has been found that neutrophils are also important participants in tumorigenesis and development. In TME, neutrophils accumulate in large numbers, and cancer cells are involved in regulating neutrophil recruitment and changing the phenotype of some neutrophils to TANs (Figure 1). Based on the phenotype and function of neutrophils, TANs can be classified as antitumor (N1) and protumor (N2) neutrophils, indicating that TANs are highly heterogeneous [135, 136]. Many cytokines in TME are also involved in the polarization of TANs. For example, IFN‐I, as a major cause of neutrophil polarization to the N1 phenotype, enhances adhesion, transport, phagocytosis, oxidative burst, dysregulation, and NETs formation [137]. In addition, IFN‐γ, TNF‐α, and IL‐1β and TLR agonists are also involved in the polarization of N1 TANs. While IL‐6, IL‐10, and VEGF are associated with the polarization of N2 type TANs. Moreover, TGF‐β is a major cytokine within tumors that inhibits neutrophil activity and cytotoxicity and contributes to the phenotypic differentiation of TANs toward the N2 phenotype [135].

With the development of single‐cell sequencing and spatial transcriptomics, the heterogeneity of TANs has been further revealed. Currently, we have identified TANs in the TME of a variety of human cancers, such as breast cancer, gastric cancer, ovarian cancer, sarcoma, and lung cancer [9]. Both these TANs can interact with cancer cells, and N1‐type neutrophils can induce cancer cell aging and death through phagocytosis, release of ROS and cytotoxicity (ADCC), and modulation of various TNF‐associated apoptosis‐inducing ligands (TRAILs). While N2 neutrophils promote tumor proliferation, metastasis, immunosuppression, and angiogenesis through the secretion of cytokines or chemokines, the generation of NETs, and cell–cell interactions [58, 138]. In addition, neutrophils contribute to immunosuppression and drug resistance in cancer through various signaling pathways and ultimately lead to treatment resistance in tumors [139].

Consequently, the role of neutrophils in the process of cancer development is not static, but highly dynamic and stage dependent. At different stages of tumor development, neutrophils may exhibit opposite biological functions, such as being predominantly antitumor in the early stages while shifting to protumor in the later stages [140]. This shift in effector function is based on the heterogeneity of neutrophils. For example, the two subpopulations of TANs, N1 and N2, exhibit opposite effects, and even the same subpopulation has plasticity in different microenvironments and exhibits different effects. This mechanism of dynamic development closely influences tumor development, and under this struggle, the tumor gradually evolves from an initial suppressed development to a later stage of continuous invasive migration.

### 4.1 Early Elimination Phase

**FIGURE 2:**
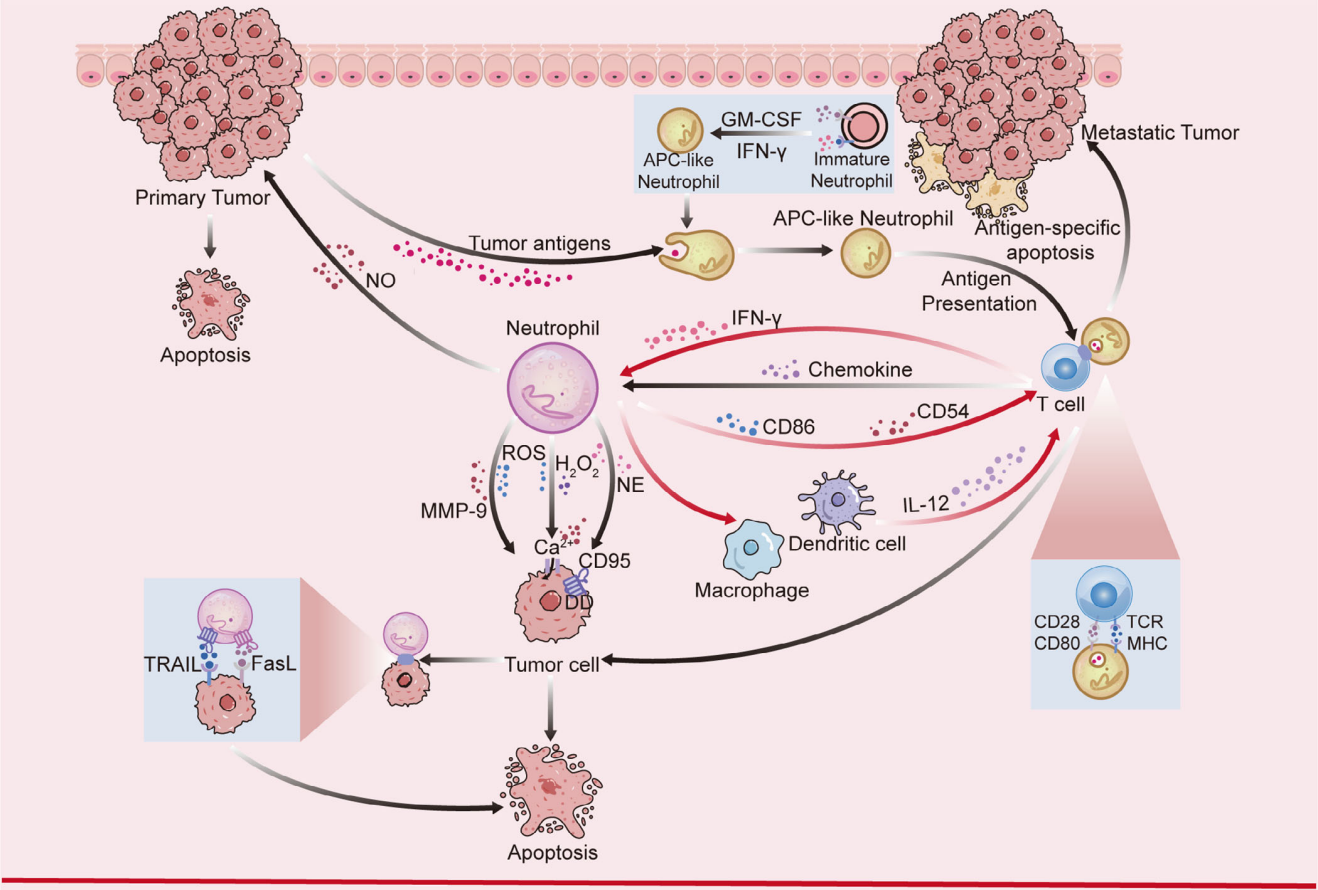
Neutrophils interact with tumor cells or immune cells to fight cancer. Neutrophils can kill cancer cells by secreting various substances such as ROS and NE or by inducing apoptosis mechanisms. Meanwhile, neutrophils can also activate and regulate immune system by interacting with T cells, DC cells, macrophages, and neutrophils that have differentiated into APCs, thereby hindering tumor progression.

In the early stages of tumor formation, such as the metastatic site or the period of small tumors, neutrophils, as important participants in the first line of defense of the body's immunity, actively inhibit tumor growth. Neutrophils are activated to participate in innate immunity, killing pathogens through various pathways such as direct phagocytosis and release of protein hydrolases, and activating adaptive immunity of the organism [141]. It has been found that N1‐type TANs can directly kill tumor cells through ADCC action and can also directly eliminate tumor cells by regulating the expression of apoptosis‐related ligands [142, 143]. Meanwhile, neutrophils can also indirectly inhibit cancer progression by activating and regulating downstream immune cells [135] (Figure 2). The metabolic mode of neutrophils at this stage still favors OXPHOS, maintaining a low metabolic state to extend survival time. Also, neutrophils rely on glycolysis to provide energy for the synthesis of ROS and bactericidal proteins and to inhibit the Warburg effect in tumors by competing for glucose.

#### 4.1.1 Neutrophils Directly Kill Tumor Cells

Neutrophils can kill cancer cells by secreting substances such as ROS, NE, or enzymes with cytotoxic effects. For example, neutrophils with enhanced expression of TRAIL and Fas ligand (FasL) can induce programmed cell death or apoptosis through direct contact with cancer cells [142, 144]. In addition, the number of neutrophils recruited in the TME is reduced due to the overall hypoxic state of the body caused by the faster metabolism of the cancer cells, but the neutrophils in the cancer environment are highly capable of killing the cells because of the high capacity of activated neutrophils to secrete ROS and MMP9, which are highly susceptible to degradation of the epithelial basement membrane [145]. Moreover, H_2_O_2_ and ROS secreted by neutrophils activate relevant Ca2+ channels on the surface of cancer cells and induce apoptosis in tumor cells by mediating Ca2+ influx into cells [60]. While IL‐17 increases the expression of TRAIL and ROS, IFN‐γ enhances the direct killing capacity of neutrophils [146]. NE secreted by neutrophils also hydrolyzes and releases the CD95 death domain, which selectively kills cancer cells and minimizes toxicity to noncancerous cells so as not to inadvertently injure normal cells [29]. And protein components in NETs, such as MPO, histones, and proteases, can exert antitumor effects and kill tumor cells, thus inhibiting tumor growth and progression [147].

#### 4.1.2 Immune Recruitment of Neutrophils

In addition to direct interaction with tumor cells, neutrophils can indirectly regulate cancer progression by activating and modulating the organism's immunity. T cells perform immunosurveillance by recognizing specific antigens expressed by tumor cells, and thus T cells play a non‐negligible role in our study of neutrophil‐regulated immunity against cancer. Some TANs have been found to activate the antitumor response of T cells, for example, Cui et al. [143] found that a specific group of neutrophil subpopulations can enhance the antigen nonspecific T cell response and tumor‐specific T cell response by expressing some signaling stimulator molecules, such as CD86 and CD54. Activated T cells can also send certain signals to neutrophils to eliminate tumor escape variants with antigenic heterogeneity and inhibit tumor progression [148]. In addition, under the influence of GM‐CSF and IFN‐γ, immature neutrophils in the TME differentiate into hybrid neutrophils with antigen‐presenting cell (APC) characteristics, which also have APC characteristics and can capture tumor‐associated antigens and migrate to the tumor‐draining lymph nodes (LNs), where they form synapses with the T cells and present the antigens to the T cells, which in turn triggers an antitumor immune response [149]. IL‐12 is a common cytokine produced mainly by dendritic cells and macrophages. Activation of IL‐12 can drive T cells to release IFN‐γ and thus exert antitumor effects. In turn, N1‐type TANs can further drive macrophages to release IL‐12, creating a positive cycle [150]. In conclusion, the interaction between neutrophils and T cells can effectively inhibit tumor progression to a certain extent.

### 4.2 Intermediate Equilibrium Phase

**FIGURE 3:**
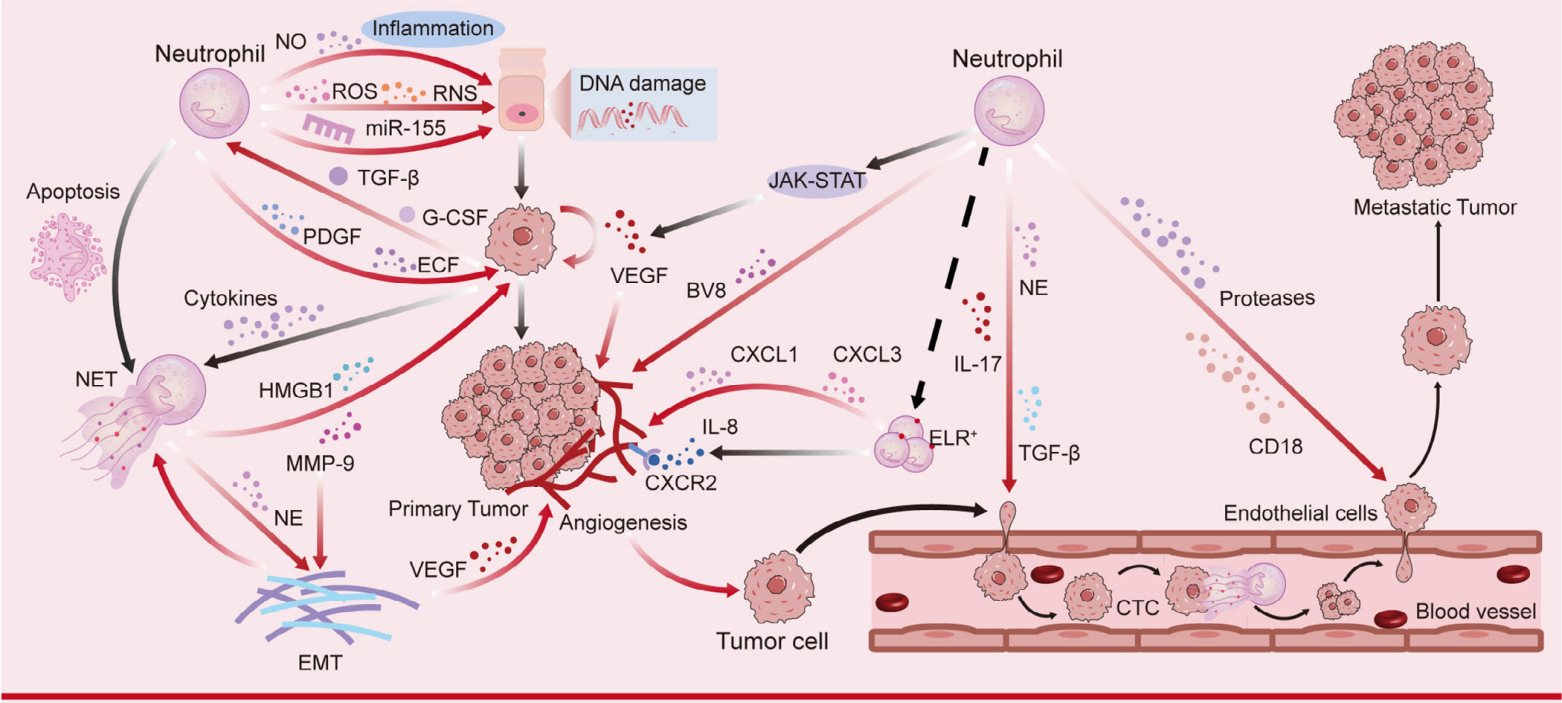
Neutrophils promote tumor growth, proliferation, angiogenesis, and metastasis. As tumors progress, the N2 phenotype of neutrophils gradually becomes dominant, promoting tumor growth and proliferation by releasing proinflammatory factors, growth factors, and NETs. In addition, neutrophils promote pathological angiogenesis through mechanisms such as promoting ECM remodeling and activating angiogenesis pathways, thereby exacerbating tumor metastasis.

In TME, chemokines (e.g., CXCL1/2) and hypoxic signals (e.g., HIF‐1α) recruited neutrophils to infiltrate, followed by an irreversible shift from an antitumor N1 phenotype driven by cytokines such as TGF‐β to a protumor N2 phenotype. Meanwhile, the N2 phenotype of neutrophils gradually dominates as the tumor grows. When exposed to tumor‐associated factors or exosomes, neutrophils undergo “functional remodeling,” which promotes tumor growth and metastasis through the promotion of immunosuppression, pathological angiogenesis, and EMT remodeling. With the continuous expansion of N2 subgroup and secretion of TGF‐β and other chemokines, N2 subgroup further expands and forms a self‐reinforcing positive feedback, resulting in a complete imbalance of the body's “balance” that regulates tumor progression, and making it difficult to reverse the trend of tumor invasion (Figure 3).

#### 4.2.1 Neutrophils Promote Tumor Growth and Proliferation

The number and function of neutrophils vary to varying degrees in different organ tissues. Dysregulated neutrophil recruitment in pathological conditions can lead to persistent infiltration of neutrophils, which can promote carcinogenesis by secreting cytotoxic proteases and oxidants that cause tissue damage and disrupt tissue integrity. A study observed that neutrophils aggregated and released large amounts of NO in infected mice's colon, accelerating intestinal inflammation and cancer formation [151]. In addition, harmful substances such as ROS released by neutrophils damage cellular DNA, while secreted proinflammatory miRNAs cause double‐strand breaks in cellular DNA and promote carcinogenesis [138]. Besides, as inflammatory cells, neutrophils can also secrete a variety of growth factors to directly promote the proliferation of tumor cells, including platelet‐derived growth factor and epidermal growth factor. And neutrophil‐derived NETs and their associated molecules MMP9 and HMGB1 can also induce cancer cell proliferation [152, 153, 154]. In addition, it has been shown that NETs can induce inflammation in the TME by promoting ECM remodeling and increasing vascular permeability, creating a procancer environment that exacerbates tumor progression [155].

Similarly, cancer cells also regulate the expression of cytokines in the body through multiple pathways, which in turn affects neutrophil recruitment and contributes to the protumor role of neutrophils. For example, G‐CSF, as an important stimulator of neutrophil release, promotes the mobilization of neutrophils from the BM to the circulatory system and also prolongs the lifespan of neutrophils through inhibition of apoptosis, allowing for an expanded role, whereas tumor‐derived TGF‐β in the TME promotes the polarization of TANs into N2‐type TANs [153, 156]. While induction of apoptosis attracts the release of chemokines from a large number of neutrophils and promotes neutrophil phenotypic shifts, the death of the tumor cells themselves is also one of the pathways that promote cytokine secretion.

#### 4.2.2 Neutrophils Promote Tumor Angiogenesis

Angiogenesis can provide nutrients to tumor cells and help excrete metabolites, which is important for tumor growth and metastasis, and pathological angiogenesis is also an important hallmark of cancer. Neutrophils can promote tumor angiogenesis by releasing Bv8 and VEGF and also by activating the JAK–STAT pathway, which induces VEGF expression [157, 158, 159]. In addition, ELR‐positively expressing subpopulations of TANs can promote tumor angiogenesis by directly binding to the receptor CXCR2 expressed on tumor vessels or indirectly recruiting leukocyte subpopulations, and ELR+ chemokines such as CXCL1–3 can also promote angiogenesis, and CXCR2 itself is an important factor in promoting the release of neutrophils, so the ELR+ neutrophil subpopulations are important in promoting tumor angiogenesis [160, 161].

#### 4.2.3 Neutrophil Metabolic Reprogramming

Hypoxia and metabolic changes are hallmark features of tumors [162, 163]. Tumor cells are predominantly metabolized by the Warburg effect, leading to a massive accumulation of lactate in the TME in a hypoxic environment. Nutritional levels, oxygen content, presence of signaling factors, and interference from neighboring cells cause changes in neutrophil metabolism. To maintain effector functions, neutrophils need to expend large amounts of energy and rapidly adapt to the metabolic environment of the tumor. Thus, under cancer conditions, TME induces a shift in the primary metabolism of neutrophils toward anaerobic glycolysis as an adaptation to the hypoxic environment and compensatory production of nutrients [145]. Secondary metabolism includes mitochondrial alterations, fatty acid, and amino acid metabolism, and so on, and these changes are collectively referred to as metabolic reprogramming of neutrophils [164]. HIF‐1α prompts neutrophils to adapt to hypoxic stress and promotes anaerobic glycolysis by inducing the anaerobic glycolytic enzyme PGK and activation of the NF‐κB pathway [165].

In addition, the hypoxic environment of TME further promotes anaerobic glycolysis in neutrophils, thereby exacerbating lactate production. It has been found that lactate causes immunosuppression of CD71^+^ T cells through histone lactylation and regulates neutrophil ARG1 expression, which in turn depletes arginine and hinders T cell proliferation [166]. Wang et al. [167] experimentally proved that lactate accumulation leads to the expression of IFN‐γ and TNF‐α in T cells and inhibits T cell proliferation. Lipid metabolism similarly affects neutrophil function in TME. For example, fatty acid transport proteins (FATPs) are membrane‐bound proteins that promote cellular uptake of long‐chain fatty acids and other lipids [168]. And FATPs are associated with prostaglandin E2 (PGE2) synthesis. It has been found that the PGE2–EP2/EP4 signaling axis drives neutrophil reprogramming. Inhibition of PGE2 signaling reduces the immunosuppressive activity of neutrophils and attenuates lung metastasis of breast cancer [169].

#### 4.2.4 Neutrophils are Involved in the Induction of Immunosuppression

Glycolysis in cancer cells produces large amounts of lactic acid, which tends to acidify the TME, and neutrophils and macrophages under these conditions tend to become protumorigenic, creating an immunosuppressive environment. The formation of an immunosuppressive environment is important for tumor metastasis. NK cells have natural killing activity and can kill tumor cells and infected lymphocytes nonspecifically. Some studies have found that the interaction between neutrophils and NK cells can promote breast cancer metastasis [170]. As for B cells, neutrophils can promote tumor immune escape by secreting TNF‐α, which binds to CXCL13 or CXCL12 and mediates B cell chemotaxis [171].

**FIGURE 4:**
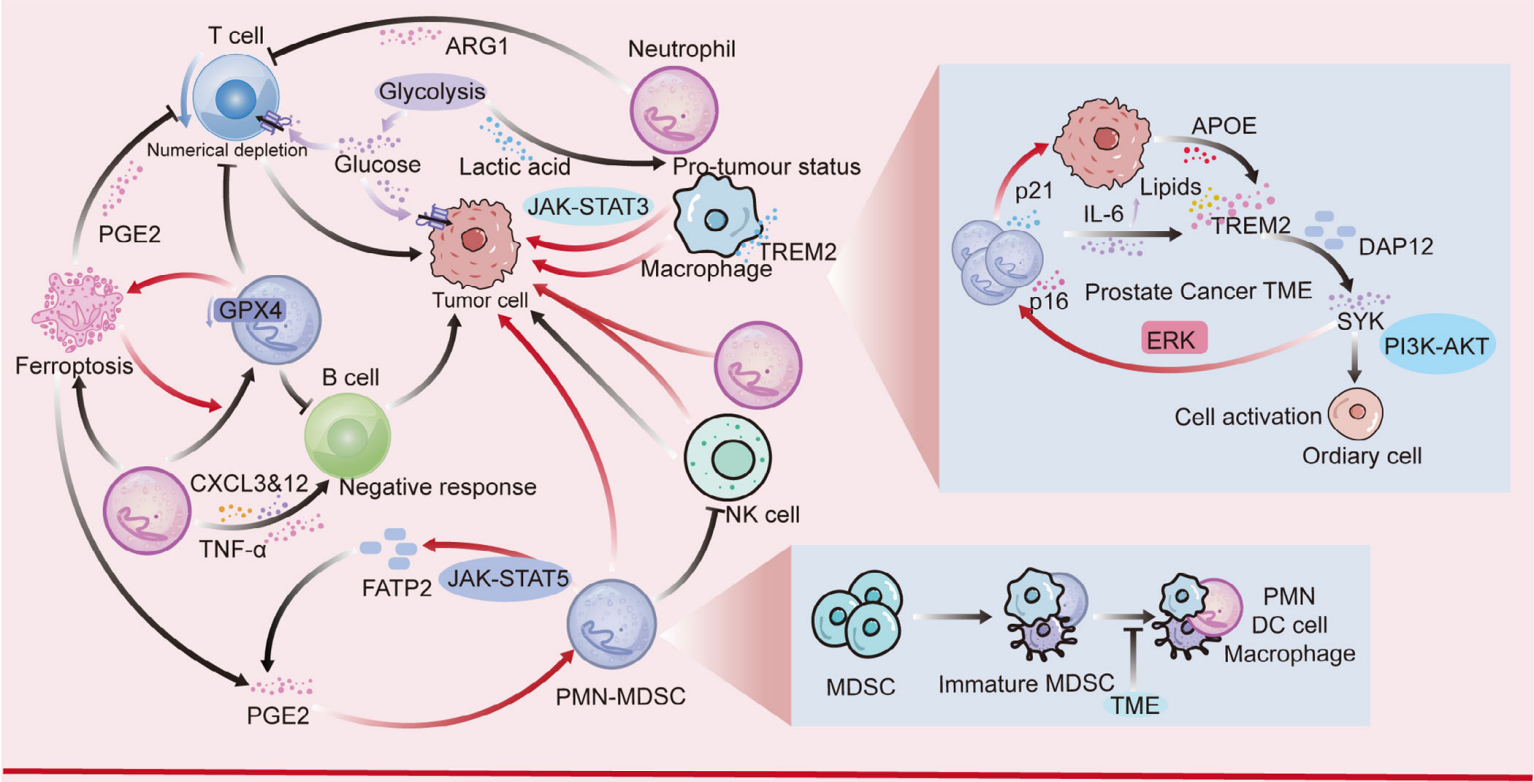
Neutrophils are involved in tumor immunosuppression. Neutrophils induce immune suppression by inhibiting the activity of various immune cells, such as T cells, B cells, and DC cells. PMN–MDSCs, which are formed by the abnormal differentiation of MDSCs, can directly inhibit the function of NK cells and promote tumor immune escape. They can also inhibit the body's immune system through the secretion of ARG1, lipid metabolism, and other nonpathways, thereby promoting tumor progression.

T cells, as a central player in cellular immunity, are essential in tumor immunosuppression (Figure 4). It has been found that neutrophils can inhibit the activation of a variety of immune cells, including DC cells, T cells, and B cells, and promote tumor immune escape [172]. Meanwhile, tumor cells compete with T cells present in large numbers in the TME for glucose uptake, leading to T cell depletion, which in turn indirectly inhibits T cell immunoreactivity and cytotoxicity, and interferes with their recruitment, remodeling, and control of their direction of differentiation, promoting T cell immunosuppression. In addition, tumor‐induced neutrophils are involved in oxidative mitochondrial metabolism, maintaining ROS production and inducing T‐cell apoptosis and suppressing T‐cell immune responses [173, 174]. Tumor‐produced GM‐CSF activates neutrophils and promotes PD‐L1 expression through the JAK/STAT3 signaling pathway. Activated neutrophils significantly suppress T‐cell immunity, while blocking PD‐L1 on these neutrophils reverses this effect [175]. Moreover, NETs are a physical barrier, they can limit the contact between cancer cells and NK or T cells [176].

In TME, there is also a special type of neutrophils, PMN–MDSCs, which are immature neutrophils formed by abnormal differentiation of myeloid‐derived immunosuppressive cells (MDSCs) under pathological conditions (e.g., tumors). PMN–MDSCs have long been recognized as an “immunosuppressive subtype” of neutrophils with potent immunosuppressive functions [177, 178]. PMN–MDSCs can inhibit the functions of T‐cells, B‐cells, and NK‐cells and also promote the tumor growth and metastasis through nonimmune mechanisms, and are associated with poor prognosis in cancer patients [177, 178, 179]. In addition, PMN–MDSCs can accelerate the depletion of l‐arginine and cysteine in TME through the secretion of ARG1, resulting in reduced CD3ζ chain expression and reduced IL‐2 and IFN‐γ production, leading to blocked T‐cell proliferation, resulting in T‐cell depletion, and consequently, inhibition of antigen‐specific T‐cell responses [178]. PMN–MDSCs can also inhibit M1 macrophage polarization and promote M2 macrophage polarization through intercellular communication, which promotes immune responses [180]. Furthermore, PMN–MDSCs inhibit IL‐12 expression in DC cells by producing IL‐10 and simultaneously impair the killing of NK cells [181, 182]. Most cancers are sensitive to iron apoptosis, and prior studies have shown that iron apoptosis is strongly associated with immunosuppressive effects in PMN–MDSCs [183]. Iron apoptosis induces the release of oxygenated lipids and releases immunosuppressive molecules such as PGE2 thereby limiting T cell activity [184]. At the same time, induction of ferroptosis also promotes the conversion of normal PMNs into immunosuppressive PMN–MDSCs, forming a positive cycle [183]. In addition, it has been found that PMN–MDSCs can intervene in tumor progression through lipid metabolic pathways. For example, PMN–MDSCs can upregulate FATP2 expression through activation of the STAT5‐related pathway, which in turn enhances the inhibitory activity of PMN–MDSCs by increasing arachidonic acid (AA) uptake and PGE2 synthesis, and ultimately supports tumor growth and promotes immune escape [185]. Moreover, oxidized AA–PE accumulated during iron apoptosis may also have a direct inhibitory effect on T cells, which would complement the role of PGE2. In conclusion, during tumor progression, neutrophils combined with PMN–MDSCs promote T‐cell depletion, TME imbalance, and immunosuppression through their unique immunosuppressive function, which further contribute to tumor escape and become an important driver of tumor growth and metastasis.

### 4.3 Late Prometastatic Phase

In the premetastatic microenvironment (e.g., liver, lung), neutrophils become the key drivers of tumor metastasis. Tumor metastasis requires not only specific genetic characteristics of tumor cells that enhance metastatic ability, but also changes in the distant local microenvironment. Before tumor cells metastasize to the target organ, the primary tumor induces the formation of a “premetastatic niche” [186]. Kaplan et al. [187] showed that VEGFR1^+^ hematopoietic progenitor cells in the BM accumulate and form cell clusters in the premetastatic lungs. And when VEGFR1 function is blocked, premetastatic ecological niches do not form in mice [187]. Tumor cells induce a phenotypic shift of neutrophils in the premetastatic niche. For example, tumor cells can cause neutrophils in the premetastatic niche to transform to N2 by secreting IL‐6, IL‐10, G‐CSF, and TGF‐β into the premetastatic niche or by inducing cells in the premetastatic niche to produce these chemokines [186]. When neutrophils accumulate in the premetastatic niche, they change their polarization state, from inhibitory to prometastatic, leading to tumor metastasis [188].

Cytokines, enzymes and NETs produced by neutrophils can also be mediators that promote cancer cell invasion, such as IL‐17, TGF‐β, and NE. It has been found that caspase‐G and TNF secreted by neutrophils can induce cancer cell migration, and caspase‐G has the effect of hydrolyzing the ECM, which can increase the flexibility of cancer cell movement, thus enabling the tumor to gain invasive ability [152, 189]. And the elevation of a series of prometastatic factors in neutrophils, such as S100A8, S100A9, Bv8, and MMP9, can also promote tumor colonization and growth at the metastatic site [190]. In addition, leukotrienes secreted by neutrophils can also promote the metastatic and proliferative capacity of tumor cells [191]. Chemokines such as CXCR4/CXCL12 can also promote cancer metastasis by affecting neutrophil migration. Some studies have found elevated levels of CXCL12 in premetastatic ecological niches such as liver, bone, lung, brain, and LNs. Yu et al. [192] found that TNF‐α‐activated stromal cells promoted breast cancer metastasis through CXCR2‐dependent recruitment of neutrophils. And Muller et al. [193] found that inhibition of CXCR4/CXCL12 interaction in vivo significantly inhibited regional LN metastasis and lung metastasis in breast cancer. In addition, it has been found that NE, MPO as well as MMP cleave vascular endothelial adhesion proteins, leading to impaired endothelial integrity, which in turn leads to vascular leakage and promotes tumor cell metastasis [194]. Neutrophils also activate lipid carrier protein 2 (LCN2) secretion through enhanced STAT3 and induce mesenchymal–epithelial transition (MET) in cancer cells, acquiring an invasive phenotype, thereby promoting cancer cell colonization and metastasis [195].

NETs also play an important role in tumor metastasis. It has been found that NETs can trap circulating tumor cells (CTCs) by aggregating in the blood circulation or in the premetastatic niche of tumor cells, and the elevated level of CTCs facilitates tumor metastasis [196]. Neutrophils anchor CTCs to the endothelium of target organs through a contact‐dependent mechanism mediated by integrins on neutrophils binding to ICAM‐1 on tumor cells [197]. β1 integrin (ITGB1) is an integral component of NETs, and a series of in vitro and in vivo experiments have revealed that ITGB1 is expressed on both CTCs and NETs. ITGB1 can mediate the adhesion and extravasation of CTCs and NETs at the metastatic site through protein–protein interactions, promoting tumor metastasis [198, 199, 200]. Besides, NETs also can trigger the sequential upregulation of a series of genes encoding inflammatory mediators, such as COX2, IL‐1α/β, CSF‐1, and so on, which in turn can trigger tumor inflammatory response and promote tumor metastasis through the activation of the pathway represented by the TLR4/9–COX2 axis [201]. It has been demonstrated that TLR9 is a cellular DNA receptor that promotes colorectal cancer (CRC) cell proliferation, migration, or invasion by activating the MAP kinase pathway, among others [198]. And Tohme et al. [198] proposed that HMGB1 released by NETs contributes to the activation of TLR9. More importantly, NETs induce the generation of proinflammatory cytokines such as IL‐8, IL‐6, and TNF‐α, which further increase neutrophil recruitment and NETs formation, thereby promoting tumor immune escape and accelerating the process of metastatic invasion of tumors [176, 196, 199, 202]. NETs also induce tumor cells to undergo EMT, which promotes cancer metastasis. And EMT also promotes the generation of NETs. It has been found that the spontaneous formation of NETs by neutrophils in a mouse breast cancer model is enhanced, and this phenomenon is associated with the driving of EMT remodeling and cancer metastasis [203].

Neutrophils can construct immune microenvironments with other cellular components that promote cancer metastasis through a variety of interactions, and thus these interactions are considered to be immunosuppressive in their totality. For example, lung adenocarcinoma can remotely stimulate osteoblasts expressing osteocalcin in bone to provide tumor cells with N2 TANs and promote tumor progression [204]. In summary, neutrophils promote tumor metastasis by interacting with various cells and cytokines in the TME.

### 4.4 Therapy Resistance Phase

With the intervention of tumor therapies such as chemotherapy, radiotherapy, targeted therapy, or immunotherapy, the invasive progression of tumors has been inhibited. However, with the removal of sensitive cells at the initial stage of treatment, surviving tumor cells activate prosurvival pathways such as NF‐κB and HIF‐1α through epigenetic modifications (e.g., histone deacetylation), which in turn affects tumor drug resistance [205]. In addition, as neutrophils are polarized to N2 phenotype in TME and secrete various proangiogenic factors such as VEGF, accelerating the abnormal proliferation of tumor vasculature, leading to decreased efficiency of delivery of chemotherapeutic agents and so on. At the same time, the remodeling of the immunosuppressive microenvironment leads to enhanced adaptation of tumor cells, ultimately leading to therapeutic resistance [206]. The emergence of tumor drug resistance is a major obstacle to cancer treatment resistance, especially for patients with advanced or metastatic tumors. During tumor treatment resistance, neutrophils influence tumor drug resistance mainly by releasing cytokines and forming NETs, which in turn regulate apoptosis, inducing an immunosuppressive microenvironment and destroying tumor antigens [206, 207].

It has been found that neutrophils can block the efficacy of targeted therapies by activating various signaling pathways [208]. For example, neutrophils activate the protein tyrosine phosphatase (SHP2)/PI3K/Akt pathway through the release of IL‐6, which blocks dexamethasone‐induced apoptosis in human multiple myeloma (MM) cells [209]. While neutrophils in BM of MM patients protect tumor cells from doxorubicin (DOX)‐induced apoptosis [210]. In addition, IL‐6 inhibits caspase‐3 activity and hinders cisplatin and paclitaxel‐induced apoptosis in ovarian cancer cells [211]. Bevacizumab treatment enhances tumor infiltration by neutrophils and increases S‐protein expression in neutrophils, contributing to neutrophil infiltration into tumors and increasing S100A4 expression in glioma cells, interfering with the efficacy of bevacizumab to promote tumor progression [212]. Furthermore, it has been found that a subpopulation of PMN–MDSCs can induce CD8+ T cell exhaustion and CRC resistance to immune checkpoint inhibitors (ICIs) via THBS1 [213]. The HGF/c‐MET pathway was found to be an important signaling pathway mediating cancer cell–TME intertwining. And LDNs can mediate primary resistance to ICIs in non‐small cell lung cancer (NSCLC) by activating the HGF/c‐MET pathway [214]. Neutrophils can also promote tumor therapeutic resistance by mediating loss, mutation, and destruction of tumor antigens or modulating tumor antigen presentation. For example, neutrophils can hinder the formation of tumor antigens by regulating the c‐Myc gene or tumor‐associated miRNAs and thereby altering the genetics and phenotype of the tumor [215, 216]. As mentioned previously, ROS released by neutrophils cause DNA damage, which can drive the production of tumor neoantigens and is closely related to immune checkpoint blockade (ICB). In addition, it has been found that the efficiency of DC‐based neoantigen nano vaccines for hepatocellular carcinoma (HCC) can be improved by remodeling TANs, suggesting that the problem of tumor therapy resistance may be solved by neutrophils influencing the uptake of tumor antigens by DCs and the cross‐presentation of antigens to T cells [217].

It has already been mentioned that NETs can act as a physical barrier to impede the contact of immune cells, such as CD8+ T cells and NK cells, with tumor cells, thus aiding in the immune escape of tumor cells. It has been found that tumor cells in muscle‐invasive bladder cancer patients who do not respond well to radiotherapy can secrete HMGB1 and interact with TLR4 of neutrophils to promote the production of NETs and the survival of tumor stem cells (CSCs), thus forming a feedback loop. At the same time, NETs can also directly contact naïve CD4+ T cells through TLR4, promote Treg cell differentiation, inhibit the immune properties of T cells, promote immune escape, and ultimately lead to tumor drug resistance [218, 219]. In addition to acting as a physical barrier, NETs trigger T‐cell depletion and impede their activation via the PD‐1/PD‐L1 axis, diminishing the efficacy of ICIs and ultimately leading to resistance to checkpoint‐blocking immunotherapies [220]. Integrin‐αvβ1 and MMP9 secreted by NETs can activate TGF‐β, and the activation of TGF‐β can lead to the development of cancer cells that are subject to EMT and chemoresistance [221]. By secreting various procancer factors and releasing NETs, which in turn remodel epigenetics, regulate tumor cell apoptosis, immunosuppressive microenvironment, and destroy tumor antigens, neutrophils impair the effectiveness of chemotherapy, targeted therapy, and immunotherapy, among others, leading to an increased risk of tumor recurrence and metastasis. Therefore, tumor therapeutic resistance is an important reason for hindering therapeutic progress.

Overall, as important immune cells of the body, neutrophils play a very significant role in cancer progression. In the early stage of tumorigenesis, neutrophils act as the body's defense cells to kill cancer cells and inhibit cancer progression through phagocytosis, release of ROS, generation of NETs, and ADCC effects. However, with the evolution of time, neutrophils differentiate into the cancer‐promoting N2 subtype driven by microenvironmental factors such as TGF‐β. The N2 subtype gradually dominates, and the cancer‐suppressing effects presented and the N1 subtype are gradually suppressed. Driven by TME and various factors, neutrophils promote tumor proliferation and metastasis, angiogenesis, immunosuppression, and metabolic reprogramming. In advanced tumor stages, neutrophils gradually induce tumor resistance and thus lead to therapeutic resistance. In conclusion, neutrophils are dynamic and two‐sided in the cancer process, gradually changing from “defenders” to “accomplices” as the disease progresses. The study of neutrophils will help us to better understand the progression of cancer and explore effective therapeutic targets.

## 5 Therapeutic Targeting: From Molecular Dissection to Clinical Translation

Neutrophils play different roles in traditional treatment modalities such as radiotherapy, chemotherapy, and surgery and are also closely associated with tumor prognostic indicators. In the preceding section, we summarized the important role of neutrophils in cancer progression, and the development of neutrophil‐based tumor‐targeted treatment strategies is highly necessary for us. Meanwhile, neutrophil‐related drug delivery technologies and clinical trials are continuously advancing, further driving the clinical translation of neutrophils.

### 5.1 The Role of Neutrophils in Conventional Therapeutics

Currently, the main cancer treatments are surgery, radiotherapy, immunotherapy, drug‐targeted therapy, and other treatments. Neutrophils serve different roles in these traditional treatments above [222]. Surgery‐induced inflammation activates neutrophils, which are involved in the formation of TME and trigger the formation of NETs. The surgical procedure also has the potential to cause the shedding of cancer cells, resulting in metastatic spread of the tumor. It has been shown that radiation can induce local aseptic inflammation, leading to recruitment and infiltration of neutrophils, which are transformed into TANs, while G‐CSF can induce TANs to polarize to N1 type and play an anticancer role if it is used concurrently during radiotherapy [223, 224]. Neutropenia, a common phenomenon during chemotherapy, seriously affects the therapeutic effect and patient prognosis, and clinicians usually use recombinant G‐CSF or GM‐CSF to induce neutropenia to increase patients’ white blood cell counts and enhance their immunity during chemotherapy in cancer patients [225]. Moreover, the role of NETs in chemotherapy resistance has been corroborated [221]. And some certain combination chemotherapy therapies have been indicated to induce anticancer effects of NETs [226, 227]. These studies all suggest that different subpopulations of neutrophils or NETs may respond differently to chemotherapy. Besides, it has been reported that certain subpopulations of TANs significantly affect the efficacy of tumor immunotherapy and that effective immunotherapy can cause an increase in the number of TANs [150].

### 5.2 Neutrophil‐Associated Prognostic Indicators

In addition to the above therapeutic tools, there are many factors that influence the therapeutic effect of neutrophils. First, the intrinsic characteristics of the tumor itself, such as staging, tissue specificity, and metabolic shifts, profoundly affect the role of neutrophils, while factors such as mediator secretion and microbial aggregation in the TME are also involved in regulating the function of neutrophils. So, neutrophils always influence cancer prognosis.

Notably, the activity of neutrophils in cancer changes depending on the stage of tumor progression. It has been shown that the accumulation of neutrophils in tumors and LNs in the early stages of tumors is a better predictor of prognosis because neutrophils in this period are predominantly of N1 phenotype and have antitumor ability. In contrast, at later stages, neutrophils are reprogrammed by tumor‐derived factors and polarized to the N2 phenotype, becoming protumorigenic [149]. Overall, higher neutrophil infiltration in advanced TME has been consistently associated with a poorer prognosis, which also correlates with the predominance of N2 TANs [228].

The use of ICIs in cancer therapy has led to an increase in immune‐related adverse events (irAEs), which can lead to treatment interruption and poorer prognosis. It has been shown that the higher the neutrophil–lymphocyte ratios (NLRs), the worse the prognosis in cancer patients’ organisms [229]. There are evidences highlighting the close association between elevated levels of NLRs and activation of innate immune response, suggesting that NLRs may be important in predicting irAEs as a reflection of tumor‐driven inflammatory processes, which may help to monitor disease progression [230, 231]. And the mechanism by which increased neutrophil counts are associated with irAEs may be related to the suppression of T‐cell function by N2‐type TANs through ARG1, TGF‐β, and so on [166, 232]. And MDSCs can also synergistically inhibit the immune response through pathways such as ROS/PD‐L1.

NETs have been recognized as an independent prognostic factor for cancer, and studies have shown that the physical barrier formed by NETs can block the action of some ICIs and degrade T‐cell‐associated chemokines, thereby reducing the effectiveness of immunotherapy [176]. By quantitatively detecting NETs or derivatives of NETs such as MPO‐DNA, NE‐DNA, or Circ‐DNA, the prognosis of the tumor can be reflected a certain extent, and H3Cit, as a representative marker of chromatin depolymerization in the process of NETs formation, can also be used as a prognostic indicator for cancer patients [64, 233, 234]. In addition, NETs markers are more sensitive than conventional tumor markers such as AFP and CA19‐9, which helps in the early diagnosis of tumors.

### 5.3 Targeting Strategies Classification

In recent years, with the rapid development of biotechnology, the technology of targeted therapy for neutrophils has been advancing. Compared with traditional radiotherapy and surgery, targeted therapy can achieve therapeutic effects more efficiently and safely. Currently, there are three main directions of neutrophil‐based tumor therapeutic strategies, including (i) targeting to increase the number of circulating neutrophils and enhance their anticancer function; (ii) targeting to inhibit the function of protumorigenic neutrophils; and (iii) targeting the immune‐suppressive function of neutrophils [235]. Because of the important role of NETs in the tumor process, therapeutic strategies based on NETs are often singled out and developed as a separate narrative.

#### 5.3.1 Targeting to Increase the Number of Circulating Neutrophils and Enhance their Anticancer Function

The neutrophils referred to here are mainly conventional neutrophils and TANs polarized to the N1 phenotype. On the one hand, we can induce the release of neutrophils and prolong their survival. g‐CSF promotes a faster release of neutrophils from the BM and enhances their biological function [236]. The simultaneous use of recombinant G‐CSF during chemotherapy to alleviate neutropenia in cancer patients is a concrete manifestation of this therapy, which has already achieved some success in clinical practice [223].

On the other hand, we can promote polarization of the N1 phenotype. For example, using IFN‐γ, TNF‐α, or TLR4/TLR9 ligands to induce antitumor phenotypes. Alternatively, repolarization of N2‐type TANs to N1‐type TANs and activation of their antitumor status is also a promising therapeutic approach. For example, the phenotype of TANs can be reprogrammed by inducing N2 to N1 conversion via IFN‐γ and TNF‐α, and so on, or by combination therapy with the CXCR2 inhibitor AZD5069/anti‐PD1 [237, 238]. TGF‐β inhibitors can also shift neutrophils to the N1 phenotype [239], And the conversion of N2 phenotype to N1 phenotype can also be achieved by acidifying TME [217]. Moreover, it has been reported that small doses of salazosulfapyridine effectively inhibited nicotine‐induced polarization of neutrophils to the N2 phenotype and facilitated the shift to the N1 phenotype by reducing the expression of activated STAT3 [195]. In addition to this, there is also the idea of increasing the number of circulating neutrophils by inhibiting the CXCR4/ CXCL12 axis and thereby increasing the number of circulating neutrophils. For example, the CXCR4 inhibitor AMD3100 can mobilize HSCs, and inhibition of CXCR4/ CXCL12 signaling has been shown to be useful as an adjuvant in the treatment of hematological malignancies (e.g., leukemia) and solid tumors (e.g., breast cancer) [240, 241, 242, 243].

Furthermore, given the feasibility that reprogramming neutrophils can alter their phenotype, infusion of exogenously stimulated neutrophils to supplement in vivo neutrophil deficiencies may also be able to drive anticancer effects. It was found that exogenous infusion of immature neutrophils isolated from LPS‐treated mice was sufficient to reactivate antitumor immunity in an immune‐tolerant HCC mouse model [238]. However, exogenous granulocyte infusion is extremely risky, especially the possibility of triggering febrile reactions and pulmonary toxicity. The only disease currently permitted to be treated by exogenous neutrophil infusion is refractory neutrophilic sepsis [244].

#### 5.3.2 Targeting to Inhibit the Function of Protumorigenic Neutrophils

Since the N2 phenotype of neutrophils is protumorigenic, inhibiting the function of N2 neutrophils has also become an idea for tumor therapy. By blocking neutrophil recruitment and infiltration, neutrophils constantly tend to be depleted. Major therapeutic strategies include the use of CXCR1/CXCR2 inhibitors, C5a receptor inhibitors, selectin antagonists, anti‐integrin antibodies, leukotriene B receptor 1 inhibitors, and NETs inhibitors, aiming to neutralize neutrophil‐derived molecules, block receptor activation, and impede signal transduction [245, 246, 247, 248, 249, 250, 251].

CXCR1/CXCR2 is a potent protumor chemokine that responds strongly to IL‐8. It promotes neutrophil release and recruitment and is also associated with poor prognosis. Therefore, blocking the IL‐8/CXCR2 axis by means of CXCR2 inhibitors and other means to block neutrophil infiltration is also a promising therapeutic strategy [56, 252, 253]. CXCR2 selective inhibitors (e.g., SB225002) have shown promising efficacy in tumor therapy. It can reduce neutrophil infiltration and enhance T‐cell immunity by stimulating CD8^+^ T‐cell activation [254]. At the same time, blockade of CXCR2 can also synergistically improve the therapeutic effect of chemotherapeutic agents such as cisplatin by preventing neutrophil infiltration [254]. In addition, the use of the CXCR2 antagonist AZD5069 in HCC has been demonstrated to reverse long‐term tumor resistance to ICIs [238]. TGF‐β induces conversion of TAN to N2 phenotype. Blocking TGF‐β and targeting its downstream signaling pathways can effectively inhibit the increase of neutrophils in the N2 phenotype, thereby impeding cancer progression. Several cancer therapies utilizing TGF‐β inhibitors are currently available, including neutralizing antibodies, antisense oligonucleotides (ASOs), and small molecule inhibitors (SMIs) [255]. Treatment with ASOs or anti‐TGF‐β antibodies has been demonstrated in animal studies to delay tumor progression [256, 257, 258]. C5a produced by cancer cells can chemotaxis neutrophils and form an immunosuppressive TME by upregulating molecules such as ARG1, CTLA4, and PD‐L1 [259]. So, blocking C5a/C5aR signaling is expected to be a new anticancer therapy. In addition, SYK plays a key role in neutrophil β2 integrin and Fc receptor signaling pathways, and studies on SYK inhibitors are currently underway [260, 261, 262].

#### 5.3.3 Targeting the Immune‐Suppressive Function of Neutrophils

Due to the presence of the immune properties of neutrophils, it is also very feasible to treat tumors by targeting and enhancing the immunosuppressive function of neutrophils. Given that neutrophil‐mediated ADCC action is also an effective mechanism for killing tumors, it has also been shown that tumor cells can be eliminated by stimulating antibody‐dependent cytotoxicity in the body through artificial IgA antibodies [263]. Neutrophil‐based ICB is also a promising therapeutic strategy. By inhibiting or blocking inhibitory receptors on the surface of neutrophils to promote various antitumor functions mediated by neutrophils, which include CXCR1 inhibitors, CXCR2 inhibitors, C5a inhibitors, and NETs inhibitors as mentioned before. In addition, PD‐L1 inhibitors can effectively suppress the immunosuppressive function of TANs. Bintrafusp alfa, a dual‐action protein that inhibits TGF‐β and PD‐L1, has been validated to have favorable anticancer effects [264]. c‐MET inhibitors can target and block neutrophil recruitment and thus inhibit their functioning [265, 266]. c‐47–SIRPα pathway is an innate immune checkpoint in cancer, and blocking the interaction of CD47–SIRPα amplifies the anticancer effects of neutrophils [267, 268]. A CD47 antibody called lemzoparimab has now shown favorable cancer efficacy in the clinic [269]. In addition, activation of the STAT3 pathway leads to activation of the IL6–STAT3–PDL1 signaling cascade, stimulating tumor development and immunosuppression [270]. Currently effective STAT3 inhibitors include napabucasi, TTI‐101, and danvatirsen [271, 272, 273].

Blocking the immune mechanism of neutrophils by interfering with neutrophil‐derived molecules, blocking receptor activation, and interfering with signal transduction can also be achieved to impede tumor progression [236, 274, 275]. Some studies have found that targeted inhibition of endoplasmic reticulum stress attenuates the formation and function of PMN–MDSCs. Therefore, it is also currently being investigated to restore the antitumor activity of neutrophils by modulating MDSCs and thereby promoting their phenotypic transformation [276]. Compared with a single therapy, synergistic therapies can further amplify the effect of tumor treatment. For example, combination therapy of CXCR2 inhibitors with PD‐L1 inhibitors can amplify anticancer effects through synergistic effects [277]. And the combination of anti‐PD‐L1 therapy with Cabozantinib, a small‐molecule tyrosine kinase inhibitor that primarily targets c‐Met and VEGFR2, can also enhance antitumor efficacy [278].

#### 5.3.4 Targeted Therapy Based on NETs

NETs, as important derivatives of neutrophils, also play an influential role in cancer progression. Therefore, therapeutic strategies against NETs have emerged [91, 234, 279]. Current research on anticancer drugs targeting NETs has focused on three main areas: inhibiting the formation of NETs, removing formed NETs, and inhibiting the interaction between cancer cells and NETs.

PAD4 is an essential enzyme in the formation of NETs. It has been found that the use of PAD4 inhibitors, such as GSK484 and CI‐amidine, can effectively block the formation of NETs and inhibit the implantation and metastasis of ovarian cancer [280, 281, 282]. Meanwhile, Teijeira et al. [176] demonstrated that PAD4 inhibitors and ICIs have synergistic effects. And the novel PAD4 isoform‐selective SMI JBI‐589 disrupted neutrophil chemotaxis, inhibited primary tumor growth and lung metastasis in a mouse model, and increased sensitivity to ICIs [283]. In addition, Zhang et al. [284] found that epigallocatechin‐3‐gallate inhibited the generation of NETs, thereby suppressing colon cancer migration and invasion. The presence of IL‐1β induces NETs, and the formation of NETs triggers TGF‐β‐dependent EMT and tumor cell resistance to chemotherapy. The use of IL‐1β blocking antibodies inhibits the generation of NETs, reduces neutrophil aggregation, and decreases the number of lung metastases after chemotherapy [221]. DNase I degrades NETs and inhibits breast cancer metastasis [203]. DNase I has now been shown to be safe in preclinical trials and has been used to address the clearance of excess lung mucus caused by NETs in cystic fibrosis [201, 285]. When an adeno‐associated viral gene therapy vector expressing DNase I, AAV DNase I, is administered, it reduces neutrophil recruitment, inhibits the formation of NETs in tumor cells, and ultimately inhibits the growth of CRC metastases [286]. NE secreted by NETs can degrade the ECM, promote angiogenesis and activate procancer signaling pathways (e.g., EGFR pathway), thereby promoting tumor growth and metastasis. Inhibition of NE can block NETs‐mediated matrix degradation and slow cancer progression. GW311616, an NE inhibitor, has been shown to inhibit the proliferation of diffuse large B‐cell lymphoma cells and reduce axillary LN metastases [287, 288]. Molassamide is another NE inhibitor. It inhibits the cleavage of the NE extracellular substrates CD40 and ICAM‐1, downregulates NE‐induced ICAM‐1 gene expression, and inhibits the migration of highly invasive MDA‐MB‐231 breast cancer cells [289]. MMP9 contributes to the formation of premetastatic niches. Blockade of MMP9 effectively inhibits premetastatic niche formation and suppresses the downstream effects of NETs. The MMP9 inhibitor enalapril, when used in combination with 5‐FU, can increase the sensitivity of CRC patients to 5‐FU by synergistically inhibiting the NF‐κB/STAT3 signaling pathway [290]. In addition, the combination of NETs‐targeted therapies with existing anticancer therapies may produce synergistic effects and enhance the immune cell‐mediated anticancer effects. Thus, NETs‐targeted therapies have a promising future in cancer treatment and are expected to become a novel adjuvant tool to overcome metastasis and drug resistance.

In conclusion, current cancer therapeutic strategies based on neutrophils are mainly from the perspectives of positive regulation, negative blockade, and immune deregulation. In addition, since NETs accelerate tumor progression through mechanisms such as metastatic foci colonization, targeting NETs for degradation or blocking generation has been investigated as an independent therapeutic strategy. Targeting anticancer by precisely interfering with specific functions of neutrophils is the focus of our current research. With the continuous advancement of research, we believe that the prospect of neutrophil‐based cancer therapy will gradually improve.

### 5.4 Delivery Technology Breakthroughs

Neutrophils have a strong tropism in inflammation and cancer and are able to cross the blood–brain barrier (BBB), enhancing drug penetration and anticancer effects, as well as having low immunogenicity. Therefore, neutrophils have the potential to be used as carriers for therapeutic drug delivery and have become innovative carriers in the field of drug delivery in recent years [291]. Currently, the main neutrophil‐based drug delivery strategies include (i) neutrophil‐based carriers, (ii) biomimetic delivery systems based on neutrophil membrane‐derived nanoparticles, (iii) engineered modification of neutrophils, and (iv) combination therapy strategies [292].

Using neutrophils as a drug delivery vehicle, the researchers combined Abraxane, an albumin‐bound paclitaxel nanoparticle, with NEs to form a novel cellular drug (Abraxane/NEs). The drug can be combined with radiotherapy to induce NE homing to the tumor site, triggering NETs and simultaneous release of Abraxane, which can be internalized by tumor cells and inhibit tumor cell mitosis to achieve anticancer effects [293].

Extracellular vesicles (EVs) are generated by cells in vivo, and their low cytotoxicity and stability make them a natural vehicle for targeted drug delivery [294]. And EVs generated by neutrophils are abundant. Researchers combined neutrophil‐generated nanoparticles with NPs containing second‐generation proteasome‐inhibiting carfilzomib, which helps to reduce CTC and inhibit the formation of early metastatic niches [295]. Neutrophil exosomes wrapped with DOX have been used to cross the BBB, providing a new strategy for inhibiting glioma [296]. In a recent study, a genetically engineered hybrid liposome was invented. The liposomes were loaded with DNase I, and CCDC25 (NETs‐DNA is a ligand for CCDC25) was embedded in the liposome membrane, thus targeting NETs. The DNase I was released and started to destroy NETs, triggered by the NETs‐associated protein MMP9 [297]. In addition, Chen et al. [298] invented a nano‐platform technology that delivers DNase I to tumor sites and metastatic niches by means of near‐infrared irradiation. They indicated that this nano‐platform could enhance cancer immunotherapy and inhibit metastasis [298]. Furthermore, Liang et al. [299] found that the effect of NETs on cancer cells could be blocked by introducing a cationic material derived from polyprotic acid. The principle of action is that the strong electrostatic affinity of the cationic material for DNA competes with the binding between CCDC25 and NETs‐DNA, thus disrupting the effect of NETs [299].

Infusion of exogenously reprogrammed immune cells, such as CAR‐T and CAR‐NK therapies, is gradually changing the treatment of hematologic tumors. As a result, some researchers have also proposed the idea of granulocyte infusion and thus the treatment of cancer. CAR neutrophils are cells generated by genetically engineering human pluripotent stem cells (hPSCs), which are phenotypically similar to hPSC‐derived neutrophils, but with greater antitumor function [300]. CRISPR–Cas9 technology can be used to knock‐in genes in hPSCs to generate CLTX‐T‐CAR neutrophils, which exhibit optimal antitumor activity against glioblastoma [300]. Research on CAR neutrophils is still in the early stages of development and has now shown promising efficacy in preclinical studies related to glioblastoma [300].

In conclusion, neutrophil‐based drug delivery vehicle strategies as well as exogenous reprogrammed immune cell infusion therapies hold great promise in inflammatory diseases and tumors. As next‐generation drug delivery technologies continue to evolve, more advanced neutrophil‐based drug delivery technologies will emerge. Although technologies such as neutrophil‐based drug delivery vector strategies still present many difficulties in clinical application, they represent an emerging therapeutic direction for disease therapies.

### 5.5 Clinical Trial Design Challenges

Many interventional therapies for neutrophil involvement in tumor progression are now in clinical trials. For example, the CXCR4 inhibitor AMD3100 increases neutrophil numbers, and the CXCR2 inhibitor AZD5069 inhibits neutrophil recruitment [253, 301]. The anti‐CD40 antibody D47‐SIRPα inhibitor IBI188 has been shown to be safe and effective in a series of clinical trials by promoting neutrophil‐mediated apoptosis in cancer cells [253]. However, due to the inherent properties of neutrophils and the complex nature of TME, researching neutrophil‐based disease therapies and translating these studies into clinical applications continues to face multiple challenges. Here, we will outline the main challenges and potential solutions. These summaries may provide some assistance for progress in this area.

First, neutrophils play a “dual role” in tumor progression, and precise strategies must be developed to specifically target neutrophils while minimizing the impact on other cells and reducing side effects. How do we distinguish and target neutrophils of a particular phenotype? The researchers found that an α1‐antitrypsin‐derived peptide was able to bind specifically to NE on activated neutrophils. Coating the nanoparticles with this peptide enables selective immobilization on activated neutrophils for localized delivery [302].

In addition, the phenotype of neutrophils in TME is influenced by multiple factors such as multiple cytokines, metabolic factors, and host immune status, and is subject to human therapeutic intervention. In clinical applications, we need to monitor phenotypic changes in real time. However, existing monitoring metrics mainly rely on biopsy or imaging markers, which are difficult to capture short‐term effects. Therefore, there is in need to develop more accurate dynamic tracking techniques based on the current ones, such as single‐cell sequencing, real‐time metabolic flux analysis, and intravital microscopy [303, 304, 305].

Neutrophils are phenotypically heterogeneous and there is still a lack of more reliable biomarkers to screen and confirm whether neutrophil therapy is effective. There is a need to develop some kind of specific tumor tissue marker (e.g., N1/N2‐related specific gene expression). Neutrophil therapy often needs to be combined with other treatments (e.g., chemotherapy, ICIs, targeted drugs, etc.) for synergistic treatment, so as to amplify the therapeutic effect and maximize the therapeutic effect with half the effort. However, there are some issues that deserve our attention, for example, chemotherapy may inhibit neutrophil function, while immunotherapy may enhance their antitumor activity. How to regulate this synergistic or antagonistic effect and make the final total effect show antitumor effect is a point worth thinking about. Researchers need to validate the feasibility of drug combinations upfront through in vitro models or preclinical studies before putting them into clinical utilization. Moreover, the response to neutrophil‐targeted therapy may vary from person to person, highlighting the need for personalized treatment. In addition to this, a topic that deserves deeper consideration is that of termination indicators for clinical therapy. The efficacy of neutrophil therapy for tumor treatment needs to be combined with traditional therapeutic endpoints (e.g., OS/PFS) and immune‐related metrics (e.g., percentage of tumor‐infiltrating neutrophils, phenotypic changes). A single efficacy assessment metric may be insufficient, and the design of composite efficacy assessment metrics may be a topic worthy of in‐depth exploration. There are also many uncertainties regarding exogenously reprogrammed granulocyte infusion therapy. First, the function of neutrophils is affected by donor age and disease state (e.g., inhibition of granulocyte function in tumor patients), which may lead to activity differences between batches. Meanwhile, neutrophils have a short half‐life and short survival time in vitro (<24 h), which makes it difficult to achieve large‐scale expansion, and currently still rely on fresh isolation techniques. These are issues of concern to researchers and deserve further exploration.

Besides that, the dosing strategy for neutrophils also needs to be further optimized. The traditional dose‐escalation method may not be applicable to neutrophil dosing. In addition, neutrophils have a short half‐life and may require high‐frequency infusion, which may increase the compliance burden on patients. Therefore, metabolic engineering is also needed to extend the functional duration of neutrophils if we want to ensure that they can function for as long as possible and reduce patient burden. A caspase–LMP–oxidant–neutrophil inhibitor plus G‐CSF (CLON‐G) has been developed by researchers [306]. With the use of CLON‐G, the in vitro survival of human and mouse neutrophils was extended to 5–7 days with the maintenance of their chemotaxis, bactericidal properties and ROS production [306]. Li et al. [307] found that through the use of a method called “μ‐blood,” they were able to capture and quantitatively compare the functional heterogeneity of neutrophils in response to bacterial stimulation in healthy donors and cancer patients, and extend the lifespan of neutrophils to 6 days.

At the same time, the safety of neutrophil therapy cannot be ignored. Targeting neutrophils can unintentionally affect other cells or antitumor neutrophils, disrupting the normal immune system and making patients more susceptible to adverse effects such as infection [308]. Excessive activation of neutrophils may cause inflammatory storms or widespread tissue damage (e.g., release of excessive ROS or proinflammatory factors). Therefore, the therapeutic dose requires extra discretion in choosing the right dose for treatment in balancing the antitumor effect with cytotoxicity, as well as combining immunomodulators (e.g., anti‐inflammatory agents) to control side effects.

Regarding preclinical models of neutrophils, most current studies on neutrophil mechanisms are based on mouse models. Current mouse models for inducing tumor formation include genetically engineered models, chemical toxicity drug models, implantation models, and humanized mouse models [309]. Given that the neutrophil function and immune environment in animal models are slightly different from humans (e.g., different phenotypic markers), we may need to further validate efficacy using organoid models or more effective and innovative preclinical models.

In conclusion, clinical trials related to the targeting of neutrophils for cancer therapy face multiple opportunities and challenges. Current challenges include precision targeting, dynamic monitoring, efficacy metrics, delivery strategies, cytotoxicity, preclinical models, and the need for individualized therapy. Future studies aim to optimize targeting strategies and combination therapies, while improving monitoring metrics and exploring the potential of neutrophils as drug delivery vehicles. It is believed that the effectiveness of neutrophil‐targeted therapies will be further improved with further research.

## 6 Neutrophils Beyond Cancer: Lessons From Other Diseases

**FIGURE 5:**
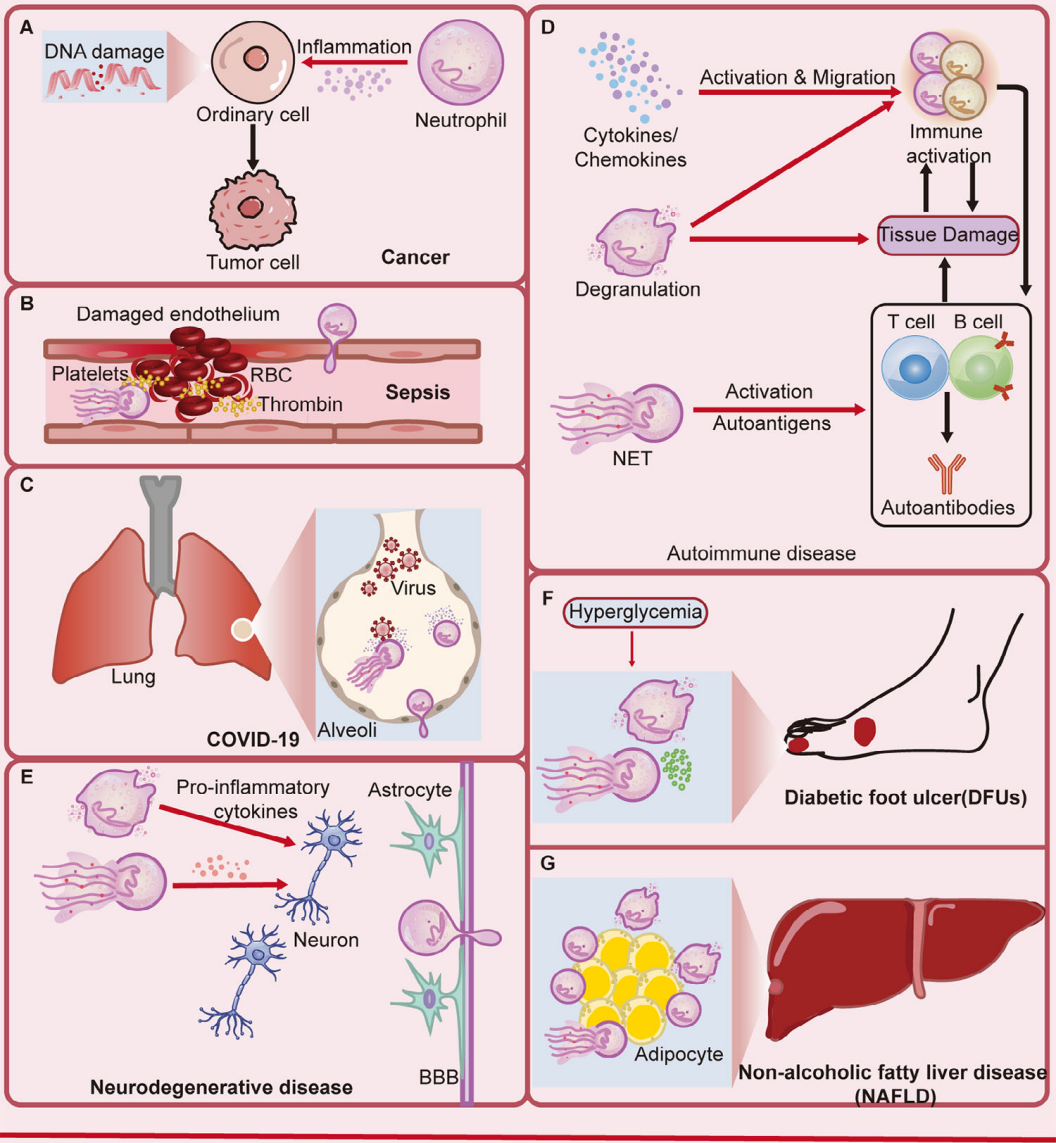
Neutrophils are involved in the progression of various diseases. (A) Neutrophils participate in the occurrence and development of cancer by inducing DNA damage and apoptosis through inflammatory responses. (B) Neutrophil activation leads to endothelial damage and thrombosis, exacerbating organ damage and even inducing DIC, ultimately leading to sepsis. (C) Neutrophils excessively release inflammatory factors and NETs, causing diffuse alveolar damage, contributing to the progression of COVID‐19, and even driving multiple organ failure and death. (D) Neutrophils induce immune activation and exposure of self‐antigens through phagocytosis, degranulation, and release of NETs, activating T cells and B cells to promote the production of autoantibodies, causing and exacerbating tissue damage, and ultimately leading to autoimmune diseases. (E) Neutrophils penetrate the blood–brain barrier (BBB), activate inflammation, exacerbate tissue damage, and promote the progression of neurodegenerative diseases. (F) A hyperglycemic environment promotes the expression of neutrophils and NETs, promotes the progression of diabetes, and accelerates a series of diabetic complications (such as diabetic foot ulcers (DFUs)). (G) Fat accumulation induces neutrophil activation and NET release, ultimately promoting the development of nonalcoholic fatty liver disease (NAFLD).

Neutrophils, as core effector cells of innate immunity, are involved in the disease process through dual roles (protective defense and pathological damage) in different diseases. In addition to cancer, neutrophils play a role in a variety of diseases such as infectious diseases, autoimmune diseases, neurodegenerative diseases, and metabolic diseases. This section provides an overview of the role of neutrophils in different diseases (Figure 5).

### 6.1 Infectious Diseases

Neutrophils specialize in defense, usually as capturing and destroying pathogens through phagocytosis, degranulation, and NETs. Activated under inflammatory or infectious conditions, they strive to eliminate inflammation and maintain body homeostasis. However, when infection and injury cannot be resolved, neutrophils are overactivated. The organism then experiences persistent inflammation and progressive deterioration, eventually evolving into an infectious disease.

#### 6.1.1 Septicemia

Sepsis is a dysregulated host response to infection that can lead to circulatory shock, multiple organ failure syndrome and ultimately death. A common cause of death in sepsis is the high level of infection in the bloodstream and the range of complications it causes. Some studies have found that neutrophils and EG drive sepsis development [310]. Neutrophil activation leads to the production of NETs. Large amounts of NETs and their products lead to excessive inflammation and cause damage to the organism. Correlation assays revealed elevated levels of NETs‐related markers in sepsis patients, further suggesting that NETs are involved in the inflammatory progression of sepsis [311, 312]. Excessive release of NETs increases vascular permeability and promotes neutrophil infiltration of tissues by destroying the ECs of blood vessels. NETs also induce a shift of EC toward proinflammatory and procoagulant phenotypes [313]. In turn, IL‐8 produced by EC further activates NETs, thus creating positive feedback and exacerbating organismal damage. In addition, innate immune system‐activated cells such as neutrophils and monocytes trigger the coagulation cascade, leading to the formation of blood clots, also known as immunothrombosis, mainly in the microcirculation [314]. NETs lead to the overproduction of thrombin and even induce disseminated intravascular coagulation (DIC), which can disrupt the microcirculation [315]. It has been shown that NETs‐platelet‐thrombin interactions promote intravascular coagulation in sepsis‐induced organ damage [316]. And histones, as an important component of NETs, aggravate microcirculatory thrombosis and exacerbate organ damage. Under the effect of multiple factors, the organ damage keeps increasing and eventually induces sepsis, even leading to death.

#### 6.1.2 COVID‐19

COVID‐19 is a clinical syndrome caused by severe acute respiratory syndrome coronavirus 2 infection. Patients may present with a range of symptoms that primarily affect the vascular endothelium and are characterized by acute lung injury, including pneumonia and acute respiratory distress syndrome (ARDS) [317]. Elevated levels of neutrophils and their associated inflammatory factors were observed in patients with COVID‐19 and were found to increase with more severe disease, suggesting that neutrophils may be involved in the progression of COVID‐19 [318]. COVID‐19‐associated pneumonia is characterized by macrophage and neutrophil infiltration of the lungs, resulting in diffuse alveolar damage that is histologically equivalent to ARDS [319]. Initially neutrophils induce immunity against coronavirus invasion by degrading viral particles and facilitating antigen presentation through actions such as phagocytosis and NETs [320]. However, with the progression of the disease, neutrophils gradually become the driving force behind viral damage to the organism. In COVID‐19, NETs are an important factor in the dysregulation of immunothrombosis. Neutrophils bind tightly to fibrin clots and platelets, resulting in microthrombosis [319]. The plasma environment of COVID‐19 and the virus itself also promotes the formation of NETs and induces epithelial apoptosis, thereby exacerbating disease progression [321]. Furthermore, neutrophil infiltration of the lungs further contributes to organ damage. NETs participate in acute lung injury by inducing the release of IL‐1β from macrophages, which in turn increases the formation of NETs [322, 323]. NETs also cause endothelial damage and necrotic inflammation through complement activation, and bind with tissue factor to release thrombus to form NETs, which may lead to multiple systemic organ failure and death [324, 325]. In conclusion, neutrophils exacerbate inflammatory lung injury and microthrombosis in COVID‐19 by excessive release of inflammatory factors and NETs production, and even drive multiorgan failure and death.

### 6.2 Autoimmune Diseases

Autoimmune diseases are characterized by the inability of the immune system to distinguish between self and non‐self, resulting in a dysregulated response to self‐antigens, which causes a range of cellular and tissue damage. Neutrophils have been found to be associated with the pathogenesis of autoimmune diseases in a number of ways. Neutrophils are frequently found at sites of tissue inflammation, suggesting that neutrophil respiratory bursts and ROS generation are important risk factors for autoimmune diseases [326]. In addition, NETs also participate in the onset and progression of autoimmune diseases and coordinate various organ inflammatory responses. A variety of autoantigens targeted by adaptive immunity, such as double‐stranded DNA, histones, citrullinated peptides, MPOs, and PRTN3, are released from NETs [327]. Typical neutrophil‐associated autoimmune diseases include SLE, rheumatoid arthritis (RA), ANCA‐associated vasculitis, and antiphospholipid antibody syndrome [328].

#### 6.2.1 Systemic Lupus Erythematosus

SLE is a classic autoimmune disease characterized by disruption of self‐tolerance, overproduction of autoantibodies, enhanced IFN‐I response, and massive release of proinflammatory cytokines, resulting in widespread inflammation and damage to multiple organs, including the kidneys, synovial joints, skin, lungs, heart, and blood vessels [329, 330]. There is evidence of elevated levels of neutrophils (including LDGs), impaired phagocytic clearance, increased apoptosis, and abnormal oxidative metabolism in patients with SLE [42, 44, 331]. In patients with SLE, neutrophils release increased amounts of autoantigens and NETs have an impaired ability to clear them, thus increasing exposure to autoantigens. Neutrophils also activate T and B cells, while promoting autoantibody production through secretion of IL‐8, IL‐17, and others.

LDGs in SLE exhibit a strong IFN‐I signature. Meanwhile, the overproduction of IFN‐α and IgG in SLE collectively induces neutrophil iron metamorphosis, which further raises the levels of IFN‐α and autoantibodies, creating a vicious cycle and exacerbating disease progression [332, 333]. LDGs also induce T cells to produce Th1 proinflammatory cytokines, including IFN‐γ, TNF‐α, and lymphotoxin‐α, which perpetuate the inflammation presence [334].

NETs further enhance IFN‐I responses by stimulating the cytosolic cyclic GMP–AMP synthase–STING pathway through higher levels of oxidized gene combinations and mitochondrial DNA [44, 335]. NETs also prompt macrophages to secrete IL‐1β, IL‐18, and TNF‐α [336]. And NETs are able to induce vascular abnormalities in SLE and promote thrombogenesis by binding to platelets and fibrin [337]. NETs are also cytotoxic, with secreted MMP9 and elastase inducing endothelial dysfunction and vascular leakage [45, 338, 339]. Various NET‐derived enzymes (e.g., NOX, MPO) oxidize high‐density lipoproteins, making them atherogenic [340]. When neutrophils migrate to the kidney, they also cause lupus nephritis [341].

#### 6.2.2 Rheumatoid Arthritis

RA is a chronic progressive autoimmune disease characterized by synovial inflammation, joint damage, bone and cartilage destruction, autoantibody production, and systemic damage such as lung and cardiovascular disease [342]. Like SLE, RA is closely associated with autoantibodies, such as rheumatoid factor and anticitrullinated protein antibodies (ACPA) [343]. Large numbers of neutrophils are present in the synovial fluid and skin of RA patients. During the development of RA, antigen‐antibody complexes constantly stimulate the immune system, resulting in delayed neutrophil apoptosis and retention in the joints. Neutrophils are over‐activated by immune complexes, releasing excess ROS, granulin (including MPO, NE) and producing NETs, thereby exacerbating tissue damage at sites of RA‐associated inflammation [344]. Some of the proteins produced by neutrophils can be carbamylated by ROS and MPO, which also promotes the production of autoantibodies against these modified peptides, thereby facilitating the formation of immune complexes that activate the differentiation of osteoclasts and the resorption of bone, which progressively destroys the bone structure and leads to severe joint damage [345]. PAD4 is elevated in neutrophils, induces citrullination of proteins during the formation of NETs and promotes the production of citrullinated autoantigens [346, 347]. RA synovial fibroblasts internalize and present citrullinated peptides from NETs to the adaptive immune system, which mediates the pathogenesis of RA, ultimately leading to cartilage damage [348]. NETs‐derived MPO disrupts the cartilage matrix and promotes the citrullination of cartilage, ultimately leading to synovitis [349].

Neutrophils also interact directly or indirectly with various immune cells in the synovial microenvironment to activate innate and adaptive immune responses, leading to severe inflammatory reactions and tissue damage [350]. Neutrophils induce monocyte infiltration, leading to prolonged inflammation [351]. NETs‐generated NE activates the Rab5a–NF‐κB signaling pathway and promotes the secretion of inflammatory cytokines by macrophages [352]. In addition, chronic exposure to NETs induces macrophage death through mitochondrial damage, activates innate immunity and leads to increased inflammation [353]. Neutrophils and NK cells mediate persistent joint inflammation through the synergistic action of IL‐18 and GM‐CSF. In turn, neutrophils also signal through ICAM‐1 and IL‐18, which enhances NK cell activation and promotes IFN‐γ production [350]. Neutrophils also exacerbate the Th1‐mediated autoimmune response to RA by promoting DC maturation. In addition, in synovial fluid, extensive infiltration of neutrophils exposes citrullinated histones associated with NETs, which provides continuous antigenic stimulation and promotes the production of ACPA by B cells. This leads to increased formation of NETs, wider exposure to citrullinated antigens, and stimulation of B cells to produce more ACPA, creating a vicious cycle that exacerbates inflammatory progression and organismal damage [354].

### 6.3 Neurodegenerative Diseases

The pathogenesis of the various acute and chronic diseases occurring in the central nervous system (CNS) is complex and involves interactions between the circulatory system and the brain. Under normal physiological conditions, microglia and astrocytes cooperate to maintain central immune homeostasis. Brain barriers, including the BBB, the blood–cerebrospinal fluid (CSF) barrier, and the blood–membrane barrier, restrict neutrophil entry into the brain parenchyma and CSF [355]. However neutrophils cooperate with a number of peripheral and brain‐resident immune cells and have been implicated in inflammatory diseases and hyperreactive immune signaling that can lead to increased brain barrier permeability and neuronal injury [356]. Once the central immune environment is altered, neutrophils penetrate the brain barrier and enter the CNS. Under homeostatic conditions, neutrophils promote inflammatory abatement and maintain neuronal function after counteracting damage. Neutrophils release anti‐inflammatory cytokines and undergo apoptosis and are then cleared by microglia. However, when the body is out of balance, activation of microglia and astrocytes may trigger a cytokine storm and recruit large numbers of neutrophils. Uncontrolled neuroinflammation leads to increased ROS production. Neutrophils generate NETs and continue to recruit more neutrophils into the center, which can exacerbate cell and tissue damage and may even lead to neuronal cell death and neurodegenerative disorders [357].

#### 6.3.1 Alzheimer's Disease (AD)

Alzheimer's disease (AD) is a common neurodegenerative disorder that leads to cognitive impairment, progressive memory loss, and decreased ability to perform activities of daily living. It is characterized by tangles of amyloid‐β (Aβ) plaques and neurofibrillary protein tau [358]. AD is still incompletely understood, but inflammation and neutrophils have been shown to exacerbate the process associated with AD [359, 360]. It has been demonstrated that neutrophils infiltrate the brain parenchyma, CSF, and blood vessels of AD patients. Aβ deposition activates neutrophils, prompting them to release NETs and inflammatory factors. Neutrophils induce tissue damage and exacerbate the inflammatory process by releasing MMPs, NE, CTSG, and MPO, and so on MMPs and NE are also involved in tissue degradation, which can damage the brain parenchyma [361]. Moreover, neutrophils acquire a toxic phenotype as they migrate within the CNS and approach neuronal cells, releasing harmful molecules that impair neuronal function [362].

Neutrophil hyperactivation releases large amounts of ROS and a range of inflammatory molecules, which in turn damage the vessel wall [363]. Activated neutrophils can also cross the BBB, and their adhesion can also lead to increased local inflammation as well as reduced cerebral blood flow and may lead to vascular dysfunction and possible BBB injury [364, 365]. Mature neutrophils also expressed CD177 and LFA‐1, which enhanced their interaction with CD31 and ICAM‐1 on ECs and promoted their infiltration into brain tissue [366, 367]. NETs also play an important role in AD. Aβ can induce suicidal NETs in AD through the propagation of oxidative stress. This leads to the degradation of amyloid fibrils by NE, releasing toxic oligomers that further exacerbate neuroinflammation [368]. Under inflammatory conditions, activated ECs release cytotoxicity factors such as IL‐1β and IL‐8 and TNF‐α, triggering NETs [369]. NETs further exacerbate neuroinflammation and increase Aβ and tau tangles, which exacerbate cognitive impairment and dementia [370]. NETs in vessels can also induce thrombin formation, exacerbating vascular inflammation and neuronal damage. NETs can also damage ECs by releasing nuclear proteins and proteases (NE, MMP, CG, etc.), and NE can also damage the BBB by increasing endothelial permeability and expression of ICAM‐1 on ECs [361, 371]. And the damage to the BBB also aggravates the condition of AD patients.

In conclusion, neutrophils and NETs exacerbate neuroinflammation and aggravate cognitive impairment and dementia in AD through various mechanisms. Further research on neutrophils may provide new ideas for the treatment of AD.

#### 6.3.2 Parkinson's Disease

Parkinson's disease (PD) is a common progressive neurodegenerative disorder characterized by typical movement disorders (including bradykinesia, rigidity, or resting tremor) as well as a variety of nonmotor symptoms (e.g., pain, paresthesia, depression).PD is characterized by the progressive degeneration of dopaminergic neurons in the substantia nigra compacta and the misfolded α‐synuclein (α‐syn) in Lewy bodies deposition [372]. Following neurodegeneration in the substantia nigra, dopamine depletion can be observed through the nigrostriatal pathway, which is the main cause of dyskinesia in PD [373].

It was found that reactive microglia were present in the postmortem brains of PD patients, and oligomers and fibrillar α‐syn released by neurons could act as DAMP to trigger an inflammatory response in microglia [374]. This led to the concept of PD neuroinflammation. However, as research continued to advance, it was discovered that PD is not limited to neuroinflammation centered on microglia [375, 376]. Peripheral inflammation is also involved in the progression of PD, and this process involves a variety of cytokines as well as peripheral immune cells, including monocytes, neutrophils, and NK cells [377, 378]. It has been found that neutrophils begin to increase in number and lymphocytes decrease many years before PD is diagnosed, suggesting that higher NLRs are associated with PD risk [379, 380]. Leucine‐rich repeat kinase 2 (LRRK2) is highly expressed in neutrophils and is associated with familial Parkinson's disease. During primary Parkinson's disease (iPD), LRRK2 is upregulated in blood immune cells [381, 382]. It has been shown that LRRK2 is involved in TLR signaling as well as pathogen response and clearance [383]. TLR‐2 and TLR‐4 have been suggested to be targets of α‐syn and are upregulated in the brain and blood monocytes of PD patients [384]. This will induce the production of proinflammatory cytokines and ROS and ultimately lead to neuronal death. Consequently, increases in TGF‐β1, IL‐1β, and IL‐6 have been consistently observed in the CSF of PD patients [385]. It has been found that the expression level of MMP9 is positively correlated with neutrophils, which promotes the entry of neutrophils into the BBB to invade the brain [386]. The deposition of α‐syn induces neutrophil aggregation and infiltration of the CNS along with the neutrophils, mainly targeting the substantia nigra, leading to neuroinflammation and motor symptoms [375, 387]. Activated neutrophils also release proinflammatory cytokines such as TNF‐α, which directly damage dopaminergic neurons and exacerbate dopaminergic neuron death through NETs‐mediated inflammation [388]. In addition, these cytokines promote the migration of peripheral immune cells to the brain, thereby indirectly exacerbating neuronal damage and leading to worsening of motor symptoms [389]. Also active neuroinflammation can further produce proinflammatory cytokines, thereby exacerbating neuronal damage [390].

In conclusion, although there are still insufficient studies on the role of neutrophils in the pathogenesis of PD, we have been able to determine that neutrophil alterations may be associated with neurodegeneration and contribute to the persistence of chronic inflammation. Further specific studies are needed to assess the role of neutrophils in PD [391].

### 6.4 Metabolic Diseases

Neutrophils play a key role in metabolic diseases through chronic low‐grade inflammation, metabolic tissue infiltration and immunometabolic reprogramming. The “metabolic–inflammatory axis” is a central driver of such diseases, such as type 2 diabetes mellitus (T2DM), obesity, and nonalcoholic steatohepatitis (NASH). The core mechanisms involve the release of proinflammatory factors, formation of NETs and cell–cell interactions that drive insulin resistance, lipid metabolism disorders, and vascular injury.

#### 6.4.1 Type 2 Diabetes Mellitus

T2DM is a chronic metabolic disease characterized by hyperglycemia, hyperinsulinemia, and dyslipidemia as a result of progressive insulin resistance, pancreatic beta‐cell dysfunction, oxidative stress, and chronic low‐grade inflammation [392]. As the disease progresses, T2DM can lead to the development of a variety of complications, including diabetic foot ulcers (DFUs), diabetic retinopathy, NASH, neuropathy, cardiovascular disease, and renal disease [393].

Metabolism and inflammation are highly interdependent, and the metabolic changes and inflammatory environment of T2DM result in the activation of a large number of neutrophils [394]. Patients with T2DM have elevated levels of neutrophils in vivo and rely predominantly on glycolytic energy production, exhibiting reduced chemotaxis, phagocytosis, and apoptosis, as well as increased ROS secretion, degranulation, proinflammatory cytokine production, and NETs production. These changes result in decreased bacterial clearance and increased ability to induce tissue damage [395]. It has also been shown that neutrophils play a key role in regulating insulin resistance in insulin‐sensitive tissues as amplifiers of inflammatory signals and chemotactic agents of other inflammatory subpopulations [396]. It has been found that the lack of intestinal epithelial barrier integrity in patients with T2DM results in excess granulopoiesis due to the entry of gut‐derived microbial antigens into the BM through the body circulation [395]. At the same time, the impaired intestinal barrier leads to endotoxin entry into the bloodstream forming endotoxemia. The endotoxemia will in turn activate systemic inflammation. The gut in T2DM has also been found to be a site of increased neutrophil activity, so neutrophils may be involved in damaging the body barrier.

In addition, hyperglycemia‐mediated NOX triggers elevated ROS levels and enhances the production of NETs [397]. The formation of NETs is associated with inflammation, thrombosis, endothelial dysfunction, impaired wound healing, and diabetic complications. Enhanced NETs may induce weakening of the intestinal epithelial and endothelial barriers and prevent their repair by vascular repair cells. Several studies have shown that elevated neutrophil‐derived proteases, ROS, and NETs inhibit diabetic wound healing [398, 399], and accelerate a range of diabetic complications, including diabetic retinopathy [122]. NETs also contribute to peripheral arteriopathy by blocking peripheral circulation, thereby increasing the risk of lower limb ischemia, stroke, myocardial infarction, and embolism [400]. NETs also modulate the rapid functional upregulation of the neutrophil CD11b/CD18 complex, promoting increased neutrophil adhesion to the ECM and ECs [400]. NETs also play an important role in wound healing through IL‐8/MMP9/fibroblast crosstalk that impedes wound healing and plays an important role in the progression of DFUs [401]. Topical application of the selective MMP9 inhibitor (R)‐ND‐336 has been shown to accelerate wound healing in diabetic mice with DFUs [402].

#### 6.4.2 Nonalcoholic Fatty Liver Disease

Nonalcoholic fatty liver disease (NAFLD) as a chronic liver disease is characterized by accumulation of liver fat, persistent inflammation and hepatic fibrosis. NAFLD includes simple steatosis and NASH, which is usually associated with obesity, insulin resistance, diabetes mellitus and dyslipidemia [403]. Fat accumulation in hepatic cells with inflammation and damage is associated with the risk of developing liver fibrosis, cirrhosis, or HCC [404, 405]. Studies have shown that the gut microbiota plays a key role in the pathogenesis of NAFLD. Gut vascular barrier damage driven by the microbiota leads to the entry of bacteria and their products into the circulation [406]. Hepatocyte apoptosis promotes immune cell recruitment and activates the immune system by stimulating the secretion of EVs and multiple chemokines (e.g., IL‐1β and IL‐18), while Kupffer cells (KCs) contribute to inflammation and fibrosis by activating the NF‐κB pathway, which leads to a massive release of cytokines. KCs contribute to the development of NASH through an increase in the production of TNF‐α and CCL2 in the early stages [407]. Some studies have found that neutrophils also play a key role in the development of NAFLD. NLRs are considered to be a valid diagnostic marker for NASH and end‐stage fibrosis in patients with NAFLD and elevated serum markers for NETs correlate with the severity of NASH.

Fat accumulation causes an inflammatory response, which in turn induces activation of infiltrating neutrophils and release of NETs. Increased secretion of MPO by neutrophils promotes the development of inflammation and insulin resistance and facilitates hepatic fibrosis through activation of hepatic stellate cells (HSCs) by derived IL‐17A. In addition, overexpression of CXCL1 or IL‐8 in hepatocytes promotes neutrophil infiltration and activation of the ASK1/p38MAPK pathway through massive release of ROS, which in turn promotes steatohepatitis toward NASH [407, 408]. Neutrophil‐derived IL‐22 and IL‐17 also promote the production of NETs. NETs are involved in the disease progression of NAFLD by promoting oxidative stress and inflammation and contribute to hepatic fat accumulation and liver injury. NETs can trigger additional inflammation by recruiting other immune cells to the liver to participate in the immune response. For example, NETs can promote the progression of NASH by regulating Treg cell differentiation [409]. Furthermore, NETs‐derived IL‐22 can promote cirrhosis and thus the development of HCC by activating the STAT3 pathway [410]. LCN2 is a neutrophil‐specific protein, and elevated levels of LCN2 have been found in NASH patients. LCN2 is involved in attracting neutrophils and enhancing the expression of inflammatory mediators such as TNF‐α, IL‐1β, and MCP‐1 [411]. In addition, LCN2 induces CXCR2 expression, activates the ERK1/2 pathway, and produces proinflammatory factors, enhances macrophage infiltration and neutrophil–macrophage crosstalk, thereby inducing NASH [412]. Neutrophil‐derived EVs have been found to be elevated in NASH mice and are involved in proinflammatory effects and regulation of immune function. Various proinflammatory or anti‐inflammatory miRNAs are often carried in these EVs and released in response to stimulation by proinflammatory factors or LPS [413]. The development of drugs targeting miRNAs may be beneficial for the improvement of NASH. Currently miR‐690 has been shown to reduce liver fibrosis and steatosis, which is beneficial in restoring the function of specific KCs in NASH [414]. In conclusion, neutrophils play an important role in the disease progression of NAFLD by releasing ROS, inflammatory factors, and NETs, which directly damage hepatocytes and drive the progression of NAFLD to NASH and fibrosis.

As an important player in the immune system, neutrophils play an indispensable role in the development of infectious diseases, autoimmune diseases, neurodegenerative diseases and metabolic diseases. During the disease process, neutrophils usually play the dual role of “defense first and then damage.” In the early stage of disease, neutrophils play a protective role through phagocytosis and NETs to capture pathogens or remove aberrant proteins. However, when driven by persistent inflammatory stimuli or metabolic disorders, neutrophils become over‐activated, leading to a storm of NETs, a massive release of proteases, and an amplification of proinflammatory factors, which in turn aggravate tissue damage (e.g., sepsis with multiorgan failure, diabetic vasculopathy, etc.). By studying the mechanisms by which neutrophils regulate disease progression and balancing their immunosurveillance and destructive effects, it may provide new ideas for disease treatment.

## 7 Controversies and Future Horizons

Neutrophils are the core effector cells of innate immunity, and their function is traditionally thought to be limited to pathogen clearance during acute infection (specific effects include phagocytosis, degranulation, and NETs release). In recent years, the role of neutrophils in homeostasis and disease has been further explored with the advancement of related research. Neutrophils maintain homeostasis of the organism through immunomodulation, angiogenesis, tissue repair, and metabolic regulation. In chronic diseases (e.g., cancer, autoimmune diseases, and metabolic diseases), neutrophils have complex and multifaceted roles. The effects they exhibit are closely related to their phenotype, but the mechanisms and clinical significance are still highly controversial.

It is well known that the rapid activation and relatively short lifespan of neutrophils complicate the study of their function and metabolism, as these effects can change rapidly in response to changes in the environment. Disease progression is a dynamic and changing process. It is possible for neutrophil function to exhibit completely opposite effects at different stages of disease progression. For example, in the early stages of disease, neutrophils act as “defenders” as innate immune cells. However, as the disease progresses, neutrophils may become “drivers” of the disease, contributing to its progression and jeopardizing the host organism. In the TME, neutrophils are polarized into different phenotypes due to their heterogeneity and thus have different functional and metabolic characteristics and interact with tumor cells and other cells in the TME. In the TME, neutrophils are activated to produce cytokines that promote tumor proliferation and metastasis. Immunotherapies targeting neutrophils have shown efficacy in inhibiting tumor growth and metastasis. Current neutrophil‐based cancer therapies mainly consist of activating and enhancing the antitumor phenotype of neutrophils, or inhibiting the activation and action of the protumor phenotype of neutrophils by targeting cytokines or receptors. However, due to the intricacies of the TME, strategies that simply inhibit the N2 phenotype or induce neutrophils to shift to the N1 phenotype while reducing their immunosuppressive effects are often insufficient to achieve significant clinical efficacy. Moreover, these therapies do not inherently block the polarization of cancer cells to neutrophils, which largely limits the progress of tumor therapies targeting TANs [266].

Therefore, there are still many questions that deserve to be further explored in this process. First of all, does the phenotypic polarization of neutrophils occur before the appearance of cancer cells, or is it induced by the presence of TME and thus the polarization of neutrophils? Further exploration of this question will help us to deepen our understanding of the biological role of neutrophils. In the initial stages of cancer, neutrophils play the role of “defenders.” Chronic inflammation causes neutrophils to be recruited in large numbers to recognize and destroy cancer cells more quickly. However, the reality is that chronic inflammation is more likely to cause cancer. Second, the question about how to precisely target specific subtypes of neutrophils remains a worthwhile one. Neutrophils often lack specificity in cancer therapy, and targeting neutrophils may inadvertently affect other cells or antitumor neutrophils and disrupt the normal immune system. Therefore, exploring specific markers (e.g., cell membrane proteins, cytokines, etc.) for particular subpopulations and thus achieving precise targeting appears necessary. In addition, neutrophils have a short lifespan, and when we target and block a batch of TANs in the TME, there is a high probability that the batch of TANs will be cleared before the blocking effect takes effect and be compensated by fresh TANs. Therefore, how to prolong the duration of their action by modulating the lifespan of neutrophils and thus amplify the therapeutic effect? This is also a question worth thinking about.

Therefore, the research and development of neutrophil‐based tumor therapies should focus on the development of new targeted blocking drugs that inhibit the conversion of neutrophils from N1 to N2 phenotype and precisely target specific neutrophil phenotypes, while avoiding interference with the normal immune function of normal neutrophils throughout the body and maintaining their normal function of defending against microorganisms and antitumor to achieve protective immunity and minimize the minimize collateral damage to the host organism. At present, broad suppression of excessive inflammation using nonspecific immunosuppressive agents remains the mainstream treatment for tumors. Various receptor antagonists or inhibitors are indeed effective in reducing tumor progression. However, more efforts are needed to address the broad inhibitory effects and erratic side effects of these drugs. Neutrophil‐targeted tumor immunotherapy still faces important clinical limitations, including lack of specificity, short duration of action, and complex crosstalk with other immune and stromal cells in the TME. Neutrophils are highly migratory in inflammation and are able to penetrate the BBB, thereby enhancing drug penetration and therapeutic efficacy. Drug delivery strategies targeting neutrophils in combination with nanoparticle technology are also currently advancing, and the potential of neutrophils as drug delivery carriers needs to be urgently exploited. In addition, cutting‐edge technologies such as gene editing can be utilized to enhance the activity and specificity of neutrophils, thereby improving therapeutic efficacy. Furthermore, we can also try to combine neutrophil‐targeted therapy with radiotherapy, chemotherapy, and immunotherapy to develop an individualized treatment plan for patients, in order to maximize the therapeutic effect and improve the prognosis of patients.

In conclusion, immunotherapy targeting neutrophils presents both opportunities and challenges, and how to translate existing scientific results into practical clinical applications remains a major concern.

## 8 Conclusions

Neutrophils play an important role in the homeostatic balance of the organism through their effector functions of phagocytosis, degranulation, and production of NETs, which in turn play an immune role. In addition, neutrophils are involved in various physiological functions such as angiogenesis, tissue repair and metabolic regulation. Neutrophils are also involved in a variety of disease processes. The role of neutrophils in the pathogenesis of cancer is not static but highly dynamic and stage‐dependent. For example, early in life they are predominantly antitumorigenic, whereas later in life they shift to protumorigenic, a shift in function based on the heterogeneity of neutrophils polarized into two phenotypes, N1 and N2, in the TME. Moreover, neutrophils play an important role in other diseases, such as infectious diseases, autoimmune diseases, neurodegenerative diseases, and metabolic diseases. Future studies should focus on further elucidating the role of neutrophils in specific diseases, such as disease predisposition and secondary dysregulation due to other factors. We hope that research on neutrophils can facilitate the progress of neutrophil‐targeted therapies for the benefit of more clinical patients.

## Author Contributions

Xingyu Chang and Yulin Liu contributed to the conception and design of the manuscript. Junjun Qiu and Keqin Hua analyzed and finalized the figures and table. All authors have read the manuscript and approved to the submitted version.

## Conflicts of Interest

The authors declare no conflicts of interest.

## Ethics Statement

The authors have nothing to report.

## Data Availability

The authors have nothing to report.
